# LIMK1 as a Novel Kinase of β‐Catenin Promotes Esophageal Cancer Metastasis by Cooperating With CDK5

**DOI:** 10.1002/advs.202503223

**Published:** 2025-06-06

**Authors:** Shu‐Jun Li, Zhuo‐Ran Liang, Zhi‐Chao Liu, Xue‐Ping Luo, Jun‐Yi Li, Xiao‐Mei Yu, Xuan‐Zhang Huang, Yan He, Tao‐Yang Xu, Jiao‐Jiao Xu, Shao‐Cong Peng, Yu‐Xiang Song, Yan He, Xiao‐Wan Zhuang, Can‐Can Zheng, Fan Zhang, Alfred King‐Yin Lam, Wei Dai, Ming‐Liang He, Bo Liu, Qi Zhao, Guo‐Liang Lu, Jin‐Bao Liu, Zhen‐Ning Wang, Zhi‐Gang Li, Ze‐Xian Liu, Wen‐Wen Xu, Bin Li

**Affiliations:** ^1^ State Key Laboratory of Respiratory Disease Guangdong Provincial Key Laboratory of Protein Modification and Degradation School of Basic Medical Sciences Guangzhou Medical University Guangzhou 511495 China; ^2^ State Key Laboratory of Respiratory Disease Key Laboratory of Biological Targeting Diagnosis Therapy and Rehabilitation of Guangdong Higher Education Institutes the Fifth Affiliated Hospital Guangzhou Medical University Guangzhou 510700 China; ^3^ State Key Laboratory of Oncology in South China Collaborative Innovation Center for Cancer Medicine Sun Yat‐sen University Cancer Center, The First Affiliated Hospital (College of Clinical Medicine) of Henan University of Science and Technology Guangzhou 510060 China; ^4^ Department of Thoracic Surgery Shanghai Chest Hospital School of Medicine Shanghai Jiao Tong University Shanghai 200030 China; ^5^ Department of Thoracic Surgery The Fifth Affiliated Hospital of Guangzhou Medical University Guangzhou 511495 China; ^6^ Department of Surgical Oncology and General Surgery, The First Hospital of China Medical University Key Laboratory of Precision Diagnosis and Treatment of Gastrointestinal Tumors Ministry of Education China Medical University Shenyang 110001 China; ^7^ Department of Gastrointestinal Surgery The Fifth Affiliated Hospital of Guangzhou Medical University Guangzhou 510700 China; ^8^ Cancer Molecular Pathology and Griffith Medical School Griffith University Gold Coast Queensland 4222 Australia; ^9^ Department of Clinical Oncology Li Ka Shing Faculty of Medicine The University of Hong Kong Hong Kong 999077 China; ^10^ Department of Biomedical Sciences City University of Hong Kong Hong Kong 999077 China; ^11^ Fujian Key Laboratory of Precision Medicine for Cancer Department of Thoracic Surgery Palmar Hyperhidrosis Research Institute First Affiliated Hospital of Fujian Medical University Fuzhou Fujian 350005 China; ^12^ Faculty of Health Sciences University of Macau Macau SAR 999078 China; ^13^ Auckland Cancer Society Research Centre Faculty of Medical and Health Sciences and Maurice Wilkins Centre The University of Auckland Private Bag 92019 Auckland 1142 New Zealand

**Keywords:** esophageal squamous cell carcinoma, kinase‐substrate map, LIMK1, metastasis, β‐catenin

## Abstract

Metastasis is a major cause of cancer deaths, but the underlying molecular mechanisms remain largely unknown. Esophageal squamous cell carcinoma (ESCC) is a highly aggressive cancer with poor survival, yet the key kinases driving ESCC metastasis and their biological function have not been fully discovered. Here, a kinase‐substrate map of metastatic ESCC is presented for the first time by conducting a phosphoproteomics analysis of 60 clinical specimens. By further consolidating data with CRISPR/Cas9 functional screening, LIM domain kinase 1 (LIMK1) is identified as a novel kinase of β‐catenin. The in vitro and in vivo experiments demonstrated that LIMK1 cooperates with Cyclin‐dependent kinase 5 (CDK5) to promote cancer metastasis in a phosphorylation‐dependent manner. Mechanistically, LIMK1 and CDK5 synergistically phosphorylate β‐catenin at S191, enhancing its phosphorylation and interaction with Nucleoporin 93, resulting in β‐catenin nuclear translocation and activation of key pathways in cancer metastasis. High expression of LIMK1 and CDK5 is associated with poor prognosis of ESCC patients, and the clinical and functional significance of LIMK1/CDK5‐Wnt/β‐catenin axis is also verified in esophageal adenocarcinoma, gastric cancer, and lung cancer. Furthermore, the combination of LIMK1 and CDK5 inhibitors significantly suppresses metastasis in multiple models. This work highlights LIMK1 as a novel regulatory and targetable kinase of β‐catenin, informing the treatment of advanced cancer.

## Graphical abstract

Li et al. innovatively map the kinase‐substrate network in esophageal cancer metastasis. Their study demonstrates that LIM domain kinase 1 (LIMK1) and Cyclin‐dependent kinase 5 (CDK5) synergistically increase the phosphorylation of β‐catenin, thereby activating the Wnt/β‐catenin signaling pathway to promote esophageal cancer metastasis. They uncover the clinical and biological significance of LIMK1 and CDK5 in metastasis. Furthermore, the authors propose a therapeutic strategy targeting esophageal cancer metastasis using combination therapy with LIMK1 and CDK5 inhibitors.

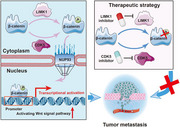

## 1 Introduction

Metastasis is responsible for over 90% of cancer‐related fatalities, reinforcing the urgent need to extensively identify effective biomarkers and explore potential therapeutic strategies. The underlying mechanisms driving cancer progression cannot be completely explained by genomic alternations.^[^ ^1^ ^]^ Protein post‐translational modifications (PTM) dramatically broaden the functional diversity of proteins. As one of the most common forms of PTM, phosphorylation has been found to impact virtually all biological functions through signal transduction, such as development,^[^ ^2^ ^]^ metabolism,^[^ ^3^, ^4^ ^]^ and innate immunity.^[^ ^5^, ^6^ ^]^ Intriguingly, only a mere 20% of kinases are accountable for phosphorylating as much as 87% of the currently annotated substrates. Over 95% of reported human phosphosites lack identification of the responsible kinases or annotated biological functions.^[^ ^7^ ^]^ Esophageal cancer is a prevalent malignancy and the seventh leading cause of cancer‐related death,^[^ ^8^ ^]^ with an overall five‐year survival rate of 22%.^[^ ^9^ ^]^ Esophageal squamous cell carcinoma (ESCC) accounts for the majority of cases, with metastasis being a main characteristic. However, there is a lack of proteogenomics studies and phosphoproteomics profiles for metastatic ESCC. A deeper understanding of phosphoproteomics and kinase‐substrate map will uncover novel mechanisms in tumor metastasis and provide important clues for cancer diagnosis and therapeutic.

β‐Catenin plays an important role in the development and progression of various tumor types, and the nuclear accumulation of β‐catenin is recognized as a critical feature for the activation of the Wnt/β‐catenin pathway.^[^ ^10^, ^11^, ^12^ ^]^ However, the key kinases and complex regulatory networks of β‐catenin remain to be elucidated and will offer great value for clinical treatment strategies. Cyclin‐dependent kinase 5 (CDK5) was initially identified as a neuron‐specific kinase due to the predominant expression of its activator p35 in postmitotic neurons. Recent studies have revealed that CDK5 is involved in several biological processes, including gene expression regulation, apoptosis,` and cell migration.^[^ ^13^ ^]^ More importantly, CDK5 binds to and phosphorylates β‐catenin and is commonly upregulated in many types of cancer.^[^ ^14^, ^15^ ^]^ Thus, it is a novel biomarker and an emerging therapeutic target in cancer treatment. However, targeting β‐catenin/CDK5 alone does not achieve the desired therapeutic effect on tumors.^[^ ^16^, ^17^ ^]^ LIM domain kinase 1 (LIMK1) is ubiquitously expressed during development and participates in many cellular processes associated with cytoskeletal structure.^[^ ^18^ ^]^ Emerging evidence indicates that LIMK1 regulates substrates other than those involved in the cytoskeleton^[^ ^19^ ^]^ and promotes tumor progression by activating the Wnt signaling pathway, correlating with cancer characteristics and poor prognosis.^[^ ^20^, ^21^ ^]^ However, there is no evidence that LIMK1 directly binds to and regulates β‐catenin, nor has a synergistic mechanism between LIMK1 and CDK5 been reported. The biological roles and regulatory mechanisms of LIMK1 in the Wnt/β‐catenin signaling pathway and cancer metastasis remain largely unknown to date.

In this study, we aimed to further expand the kinase‐substrate map, identify novel kinases that regulate tumor metastasis, and reveal their underlying mechanisms as well as clinical significance. We explored the complex regulatory mechanisms by which LIMK1 and CDK5 synergistically regulate β‐catenin nuclear accumulation and activation. Additionally, we explored the clinical and functional significance of the LIMK1/CDK5‐Wnt/β‐catenin axis in esophageal adenocarcinoma, gastric cancer, and lung cancer. Furthermore, the feasibility of combined targeting of LIMK1 and CDK5 for targeted therapy of tumor metastasis was investigated in a preclinical setting. These findings not only furnish an extensive resource to support further research in ESCC and reveal the proteogenomic features of ESCC but also propose new personalized treatment strategies for multiple cancer types, thus shedding light on the treatment of cancer metastasis.

## 2 Results

### 2.1 Phosphoproteomic Landscape of Metastatic ESCC Cohort

**Figure 1:**
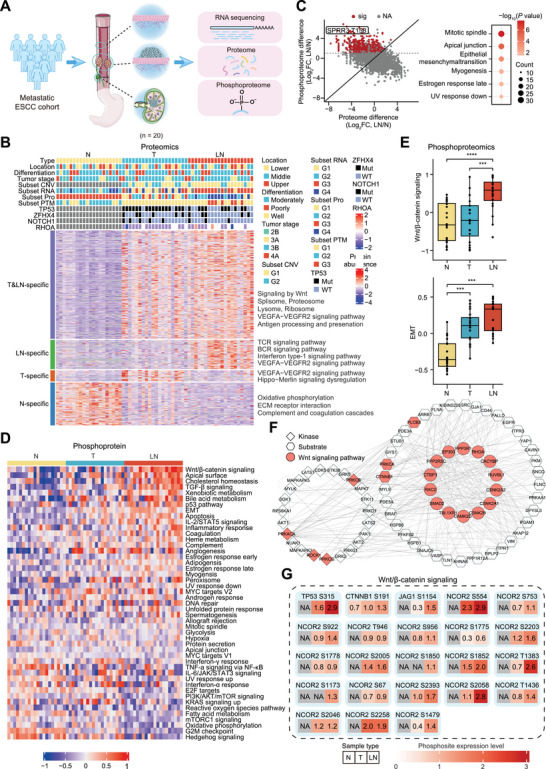
Activated Wnt signaling is a key pathway in cancer metastasis. A) Workflow of the experiment and the number of samples used for multi‐omics analysis. B) Heatmap showing the differentially expressed proteins among the four classes. The tiling bars above the heatmap show the distribution of different clinicopathological characteristics among the 20 metastatic ESCC patients. C) FC of proteins and phosphosites, and their correlations in LN and N. Pathways enriched with cancer‐related phosphoproteins. Red dots: phosphosites are greater than two‐fold changes in LN compared to N, and changes in phosphosites abundance are greater than changes in their corresponding protein abundance. D) Heatmap showing the activities of cancer hallmark‐related pathways across N, T, and LN groups. E) Boxplots showing the distribution of Wnt/β‐catenin signaling (upper) and EMT signaling (lower) among N, T, and LN groups. F) The phospho‐regulatory network links cancer‐related kinases (diamonds), phosphoproteins (hexagons), and proteins in Wnt signaling (circles). G) Phosphosites in Wnt/β‐catenin signaling were differentially expressed in N, T, and LN groups. N: normal tissues; T: tumor tissues; LN: lymph node metastatic tissues.

To obtain a comprehensive molecular understanding of metastatic ESCC, phosphoproteomic profiling was conducted based on samples from 20 ESCC patients diagnosed with metastasis, including normal tissues (N), paired primary tumor tissues (T), and matched lymph node metastatic tissues (LN) (**Figure** **1**A; Figure S1A, Supporting Information). RNA sequencing (RNA‐seq) analysis was performed to identify 17614 genes with transcripts per million (TPM) values. Using a mass spectrometry (MS)‐based label‐free quantification strategy, 7155 proteins were identified through proteomic analysis, and 23367 phosphosites were identified from 4285 phosphoproteins through phosphoproteomic analysis. High data reproducibility and technical quality were demonstrated across the entire metastatic ESCC cohort (Figure S1B–G, Supporting Information). Additionally, principal component analysis (PCA) revealed distinct differences among N, T, and LN at mRNA, protein, and phosphorylation levels, demonstrating greater heterogeneity in T and LN relative to N (Figure S1H, Supporting Information). Clinicopathological indicators are summarized in Table S1 (Supporting Information).

To investigate the changes in protein abundance across different samples, differentially expressed proteins between N, T, and LN samples were categorized into four classes (Figure 1B). The first class, defined as T and LN‐specific (T&LN‐specific), shows higher expression in T and LN compared to N samples, with LN expression higher than or equal to T. The other three classes are uniquely highly expressed in N (N‐specific), T (T‐specific), and LN (LN‐specific), respectively. Enrichment features for the four classes are shown in Figure 1B.

A phosphoproteomics heatmap displayed differential expression of phosphorylated proteins among T&LN‐specific, LN‐specific, T‐specific, and N‐specific, alongside representative pathways (Figure S2A, Supporting Information). Furthermore, 614 phosphosites (403 phosphoproteins) and 1063 phosphosites (502 phosphoproteins) were found to exhibit greater changes in T and LN compared with N samples, respectively (Figure 1C; Figure S2B, Supporting Information; *P* < 0.05, T/N or LN/N ratio > 2). These phosphorylated proteins were significantly enriched in pathways such as mitotic spindle and androgen response in T versus N (Figure S2B, Supporting Information), whereas apical junction and epithelial‐mesenchymal transition (EMT) in LN versus N (Figure 1C). The above results indicate the key regulatory role of phosphorylation modification in cancer metastasis.

### 2.2 Phosphorylation Activation of Wnt Signaling is a Key Pathway in Metastatic ESCC

To reveal key pathways regulated by phosphorylation in metastatic ESCC, gene set variation analysis (GSVA) was used to calculate pathway activities for all cancer hallmark‐related pathways. The results showed that phosphorylation is important for a series of key pathways in LN samples, with the Wnt/β‐catenin signaling pathway being the most highly regulated (Figure 1D,E). In addition, the Wnt/β‐catenin pathway signature analyzed from RNA‐seq data did not significantly differ between T and LN samples (Figure S3A, Supporting Information), further corroborating the important regulatory role of phosphorylation. To identify functionally important phosphorylation events in metastasis, a phospho‐regulatory network was constructed. The results showed that multiple signaling pathways may be regulated by a series of kinases (Figure S3B, Supporting Information). The hub proteins of the protein‐protein network were enriched in the Wnt signaling pathway, emphasizing its important role in the metastatic process (Figure 1F). The significantly altered phosphosites in the Wnt signaling pathway occurred mainly in TP53, CTNNB1, JAG1, and NCOR2 (Figure 1G; Figure S3C, Supporting Information). We subsequently examined the expression levels of downstream proteins of the Wnt signaling pathway in metastatic ESCC samples, including c‐myc, c‐jun, cyclin D1, and twist. The results showed that all these proteins were significantly upregulated in tumor tissues, particularly in lymph node metastatic tissues (Figure S3D, Supporting Information). These findings highlight the pivotal role of phosphorylation‐activated Wnt signaling in ESCC metastasis.

To systematically identify key kinases‐substrate pairs in ESCC metastasis, we performed kinase activity analysis and categorized kinases into T&LN‐specific, LN‐specific, T‐specific, and N‐specific (Figure S3E, Supporting Information). High‐confidence kinase‐substrate pairs, potentially involved in ESCC progression and therapies, were visualized, suggesting potential therapeutic strategies by targeting these kinase‐substrate pairs (Figure S3F, Supporting Information). Taken together, this phosphoproteomics data form provides additional insights into the complex molecular features of ESCC.

### 2.3 Integrated Multi‐Omics Analysis with CRISPR/Cas9 Functional Screening Identifies LIMK1 and CDK5 as Key Kinases Responsible for ESCC Metastasis

**Figure 2:**
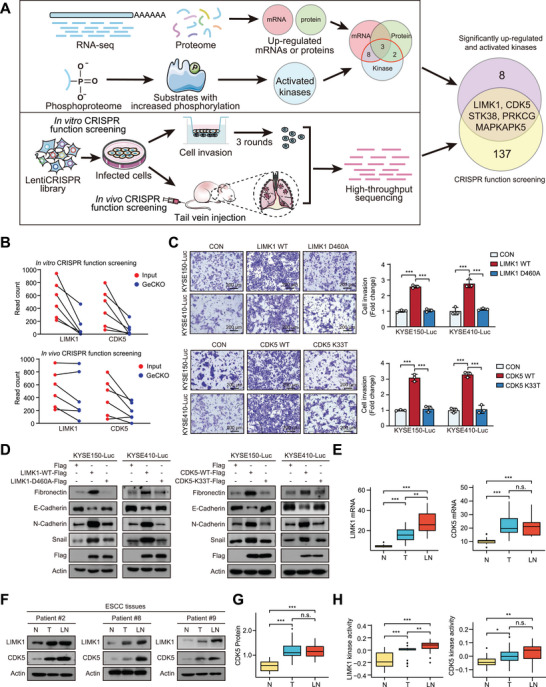
Integrated multi‐omics analysis with CRISPR/Cas9 screening identifies LIMK1 and CDK5 as key kinases responsible for ESCC metastasis. A) Diagram showing the approach used to systematically identify key kinases related to ESCC metastasis. B) Read counts of sgRNAs targeting LIMK1 or CDK5 in GeCKO‐transduced cells and input cells through in vitro (upper) and in vivo (lower) screening. C) LIMK1 and CDK5 promote ESCC cell invasion in a manner dependent on their phosphorylation activity. D) Western blot showing the effect of LIMK1, CDK5, and their mutant type on the expression levels of EMT markers in ESCC cells. E) RNA‐seq analysis of LIMK1 and CDK5 mRNA expression in N, T, and LN from metastatic ESCC cohort. F) Measurement of LIMK1 and CDK5 expression in N, T, and LN of ESCC by Western blot. G) proteomic analysis of CDK5 levels in metastatic ESCC cohort. H) Measurement of LIMK1 and CDK5 kinase activity of metastatic ESCC cohort by ssGSEA. N: normal tissues; T: tumor tissues; LN: lymph node metastatic tissues.

To systematically identify key phosphorylated kinases related to ESCC metastasis, we integrated multi‐omics analysis with CRISPR/Cas9‐based functional screening (**Figure** **2**A). Multi‐omics analysis was used to delineate kinases classified as T&LN‐specific or LN‐specific that are implicated in the metastasis of ESCC. Notably, 44 kinases were significantly activated in phosphoproteomic, while 148 and 59 kinases were significantly up‐regulated in transcriptomic and proteomic, respectively. Among these kinases, 13 overlapped in at least two omics (Figure S4A, Supporting Information). Concurrently, the GeCKO v2 CRISPR library was transduced into ESCC cells. Subsequent in vitro and in vivo screenings, coupled with high‐throughput sequencing analysis, we identified a subset of sgRNAs targeting 311 genes encoding phosphorylated kinases that were depleted, suggesting their potential role in regulating ESCC metastasis. Among these, 142 kinases had at least five sgRNA read counts that were decreased. By overlapping these 142 kinases with 13 kinases from multi‐omics analysis, five candidate kinases were narrowed down (CDK5, LIMK1, PRKCG, STK38, and MAPKAPK5) (Figure 2A,B; Figure S4B, Supporting Information). The detailed information is summarized in Table S2 (Supporting Information).

To examine which kinases promote tumor invasion, a Boyden chamber invasion assay was conducted. The results showed that overexpression of the kinases LIMK1 and CDK5 significantly promoted ESCC cell invasion in a phosphorylation‐dependent manner, compared to other candidate kinases (Figure 2C; Figure S4C, Supporting Information). Meanwhile, overexpression of LIMK1 or CDK5 did not affect the proliferation of ESCC cells (Figure S4D, Supporting Information), ruling out the possibility that their effect on cell invasion was proliferation‐based. MAPKAPK5 was excluded because it has been recognized as a tumor suppressor.^[^ ^22^ ^]^ Therefore, LIMK1 and CDK5 were selected for deeper exploration. As expected, overexpression of LIMK1‐WT or CDK5‐WT, rather than catalytically inactive mutants (LIMK1‐D460A and CDK5‐K33T), upregulated the expression of mesenchymal markers (fibronectin, N‐cadherin and snail), coupled with a downregulation of the epithelial marker E‐cadherin in ESCC cells (Figure 2D). Moreover, mRNA expression, protein expression, and kinase activity of LIMK1 and CDK5 were significantly enhanced in T and LN samples (Figure 2E–H). In addition, the datasets from TCGA also indicated that LIMK1 and CDK5 mRNA expression were significantly upregulated in esophagus cancer patients (Figure S4E, Supporting Information). These data collectively indicated that LIMK1 and CDK5 are key kinases responsible for ESCC metastasis.

### 2.4 LIMK1 Synergizes with CDK5 to Promote ESCC Metastasis

**Figure 3:**
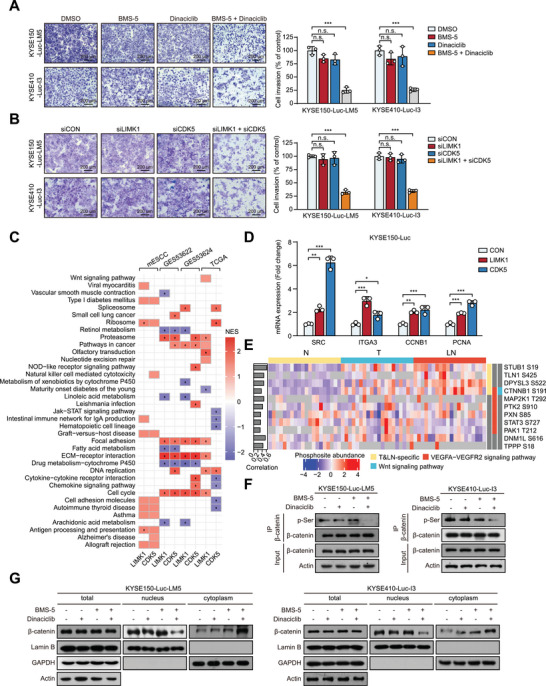
LIMK1 synergizes with CDK5 to promote ESCC metastasis. A, B) Transwell assays were performed to determine the invasive abilities of ESCC cells when treated with BMS‐5 and/or Dinaciclib (A) or subjected to knockdown via siRNA targeting LIMK1 and/or CDK5 (B). C) Transcriptome analysis of metastatic ESCC cohort and public datasets (GES53622, GES53624, and TCGA) demonstrates that the upregulation of LIMK1 and CDK5 affects multiple signaling pathways. D) RT‐qPCR analysis of SRC, ITGA3, CCNB1, and PCNA mRNA expression in ESCC cells overexpressing either LIMK1 or CDK5. E) Heatmap showing the CDK5 substrates with significantly altered phosphorylation levels in the metastatic ESCC cohort. F) The effect of BMS‐5 and/or Dinaciclib treatment on β‐catenin p‐Ser level was investigated by western blot. G) The nuclear and cytoplasmic distribution of β‐catenin was analyzed after treatment with BMS‐5 and/or Dinaciclib.

Given the important role of LIMK1 and CDK5 in tumor metastasis, the LIMK1 inhibitor BMS‐5 and CDK5 inhibitor Dinaciclib were employed to evaluate the potential of targeted therapy. Results from the Boyden chamber invasion assay showed that neither BMS‐5 nor Dinaciclib alone exhibited an obvious inhibitory effect on ESCC cell invasion (Figure S5A, Supporting Information). Interestingly, the combination of both inhibitors demonstrated significantly greater anti‐invasive effects in ESCC cells compared to monotherapy (**Figure** **3**A). Consistent results were obtained by genetically manipulating the expression of LIMK1 and CDK5 via siRNA (Figure 3B; Figure S5B, Supporting Information). To comprehensively understand the mechanisms underlying the potential synergy of LIMK1 and CDK5 in ESCC, transcriptome data from metastatic ESCC cohort were integrated with additional transcriptome cohorts from the Gene Expression Omnibus (GEO) and TCGA database. Results revealed that the upregulation of LIMK1 or CDK5 shared similar activated/inhibited signaling pathways including focal adhesion, ECM‐receptor interaction, cell cycle, and some critical signaling pathways in cancer (Figure 3C). Genes associated with pathways that were upregulated in multiple datasets in Figure 3C were selected for further validation, including RAC3 and ITGA3 in the focal adhesion signaling pathway, and PCNA and CCNB1 in the cell cycle signaling pathway. The results show that expression levels of all these genes were significantly upregulated in ESCC cells overexpressing LIMK1 or CDK5, respectively, emphasizing their potential synergy (Figure 3D; Figure S5C, Supporting Information).

Considering the crucial function of the Wnt pathway in ESCC metastasis mentioned above, the significantly upregulated β‐catenin phosphorylation at S191 drew our attention (Figure 3E). Further experiments demonstrated that combined treatment with BMS‐5 and Dinaciclib markedly reduced the phosphorylation and nuclear accumulation of β‐catenin compared to either single treatment (Figure 3F,G). These data suggest that LIMK1 and CDK5 cooperate in phosphorylating β‐catenin, promoting ESCC metastasis.

### 2.5 LIMK1 is a Novel Kinase Responsible for the Phosphorylation of β‐Catenin

**Figure 4:**
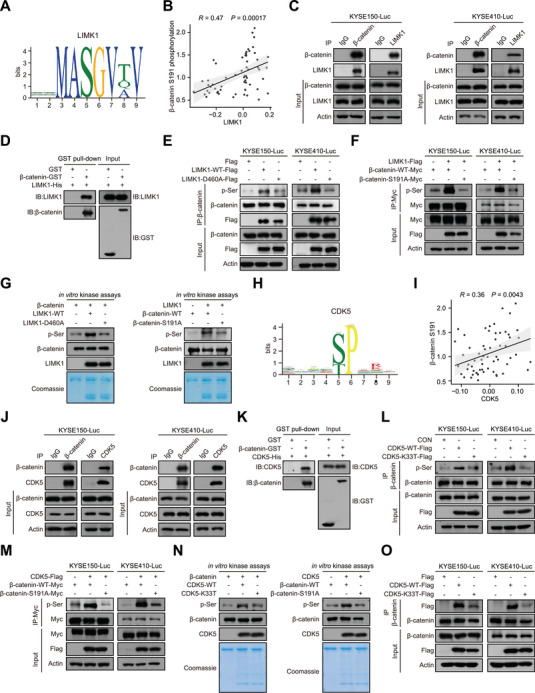
LIMK1 is a novel kinase responsible for the phosphorylation and nuclear translocation of β‐catenin. A) MEME software output showing the locations with the top‐scoring LIMK1 kinase recognition motifs. B) Correlation of phosphorylation of β‐catenin at S191 and LIMK1 expression in the metastatic ESCC cohort. C) Co‐IP assay confirming the exogenous interaction between LIMK1 and β‐catenin in ESCC cells. D) GST pull‐down assay showing a direct interaction between LIMK1 and β‐catenin. E–G) Co‐IP assay (E, F) and in vitro kinase assay (G) show that LIMK1 increases the phosphorylation of β‐catenin at the S191 site. H) MEME software output, showing the locations with the top‐scoring for CDK5 kinase recognition motif. I) Correlation of phosphorylation of β‐catenin at S191 and CDK5 expression in the metastatic ESCC cohort. J) CO‐immunoprecipitation assay was performed to confirm the exogenous interaction between CDK5 and β‐catenin in ESCC cells. K) GST pull‐down assay investigating the direct interactions between GST‐β‐catenin and CDK5‐His. L, M) Effect of the CDK5‐WT or CDK5‐K33T on the β‐catenin‐WT or β‐catenin‐S191 p‐Ser level in ESCC cells. N) Comparison of the phosphorylation level of ESCC cells overexpressing CDK5‐WT or CDK5‐K33T and β‐catenin‐WT or β‐catenin‐S191A in vitro. O) CO‐immunoprecipitation assay was performed to confirm the interaction between CDK5‐K33T and β‐catenin.

Based on the synergistic effect of LIMK1 and CDK5, we hypothesized that LIMK1 might be a novel kinase responsible for phosphorylating β‐catenin. Intriguingly, a conserved substrate recognition motif for LIMK1 was identified using MEME and FIMO software,^[^ ^23^, ^24^ ^]^ revealing that the β‐catenin S191 site is one of the sequences recognized by LIMK1 (**Figure** **4**A; Table S3, Supporting Information). Besides, there is a significant positive correlation between phosphorylation of β‐catenin S191 and LIMK1 expression (Figure 4B). Then, a series of experiments was carried out to test whether the phosphorylation of β‐catenin S191 is catalyzed by LIMK1. First, the binding of LIMK1 to β‐catenin was confirmed by Co‐immunoprecipitation (Co‐IP) and glutathione s‐transferase (GST) pull‐down assays (Figure 4C,D; Figure S6A, Supporting Information). Second, analysis of phosphorylation levels in vivo showed that LIMK1 enhanced the phosphorylation of β‐catenin S191 in ESCC cells, while LIMK1‐D460A mutant neither affected its binding to β‐catenin nor regulated the β‐catenin phosphorylation (Figure 4E,F). Third, in vitro kinase assay demonstrated that LIMK1 directly phosphorylates β‐catenin at S191 (Figure 4G). Similarly, the conserved kinase recognition substrate motif of CDK5 (Figure 4H), the correlation between phosphorylation of β‐catenin S191 and CDK5 (Figure 4I), the ability of CDK5 to catalyze the phosphorylation of β‐catenin S191 were verified (Figure 4J–N; Figure S6B, Supporting Information). Notably, the CDK5 K33 site appeared to be required for the binding of CDK5 to β‐catenin (Figure 4O). Taken together, these data suggest that LIMK1 is a novel kinase responsible for the phosphorylation of β‐catenin by collaborating with CDK5, thus playing an important role in ESCC metastasis.

### 2.6 LIMK1 Increases the Binding of β‐Catenin with NUP93 to Promote its Nuclear Translocation

**Figure 5:**
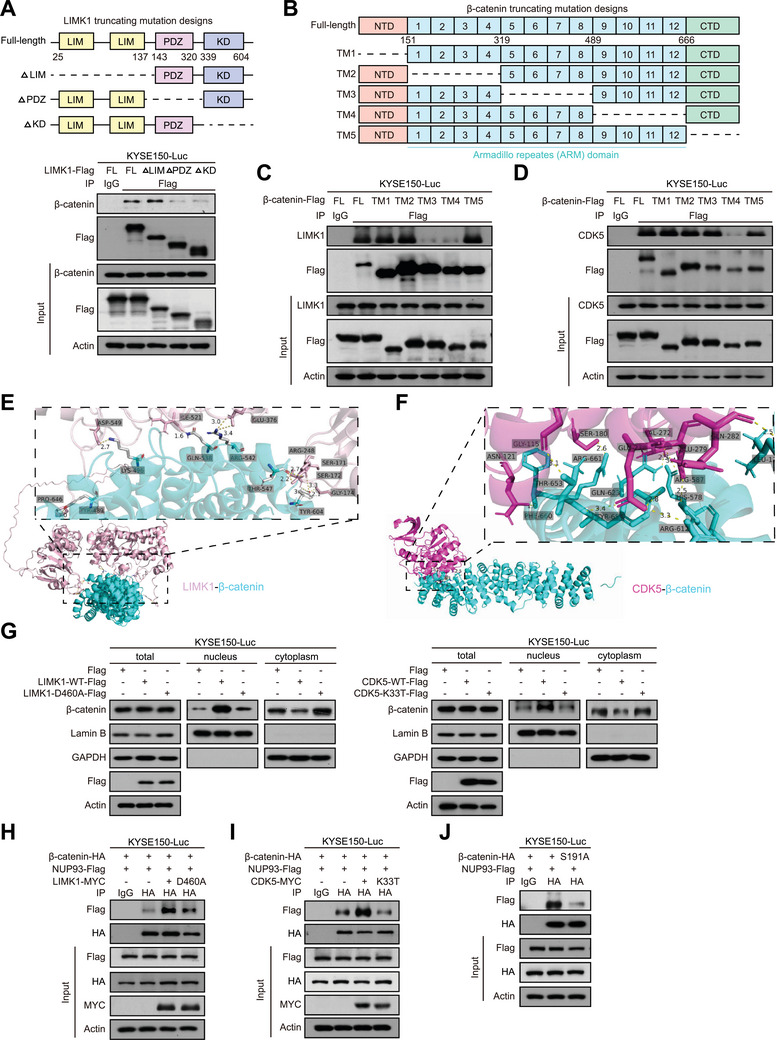
LIMK1 and CDK5 increase the binding of β‐catenin and NUP93 by phosphorylation to promote its nuclear translocation. A) Schematic diagram of the LIMK1 truncation mutants used in this study (upper). ESCC cells were transfected with LIMK1 mutants as indicated and collected for Co‐IP (lower). B) Schematic diagram of different β‐catenin truncation mutant constructs. C, D) β‐Catenin mutants were transfected into ESCC cells, and Co‐IP assays were performed to investigate the binding domains between β‐catenin and LIMK1 (C) or CDK5 (D). E, F) Predicted protein interactions based on structures of human LIMK1 (E) or CDK5 (F) and β‐catenin using ZDOCK software. LIMK1: pink, CDK5: carmine, β‐catenin: cyan. G) Subcellular fractionation was used to assess the nuclear and cytoplasmic distribution of β‐catenin in ESCC cells overexpressing LIMK1‐WT/LIMK1‐D460A or CDK5‐WT/CDK5‐K33T. H, I) Co‐IP was performed to evaluate the interactions between β‐catenin and NUP93 in the presence of LIMK1 or CDK5 in ESCC cells. J) Interaction between NUP93 and wild‐type or mutant β‐catenin was evaluated in ESCC cells.

To identify the binding region between LIMK1 and β‐catenin, a set of truncation mutants of LIMK1 was constructed, as shown in **Figure** **5**A, upper panel. The results showed that both wild‐type LIMK1 and LIMK1‐△LIM were capable of precipitating β‐catenin, whereas the interaction was not detectable upon deletion of the PDZ domain or KD domain (Figure 5A, lower panel), indicating that these domains in LIMK1 are essential for its association with β‐catenin.

Next, to identify the specific region of β‐catenin binding to LIMK1 or CDK5, five β‐catenin truncation mutant constructs (TM1, TM2, TM3, TM4 and TM5) were generated (Figure 5B). Co‐IP assays revealed that the ARM 5–8 deletion mutant of β‐catenin (TM3) and ARM 9–12 deletion mutant of β‐catenin (TM4) did not precipitate LIMK1 (Figure 5C). Similarly, TM4, the ARM 9–12 deletion mutant of β‐catenin, did not show an interaction with CDK5 (Figure 5D). We also identified the interaction domains of LIMK1 or CDK5 and β‐catenin in silico using ZDOCK^[^ ^25^ ^]^ and obtained consistent results (Figure 5E,F; Table S4, Supporting Information). These results demonstrated that both LIMK1 and CDK5 bind to β‐catenin via the ARM domain.

Till now, we are left with an incomplete picture of the intricate regulation that guides β‐catenin from the cytoplasm to the nucleus. An elevation in the nuclear localization of β‐catenin was observed upon ectopic expression of Wild‐type LIMK1/CDK5, but not the LIMK1‐D460A/CDK5‐K33T mutant (Figure 5G; Figure S6C, Supporting Information). Interestingly, nucleoporin 93 (NUP93) was documented to participate in the β‐catenin nuclear translocation,^[^ ^26^ ^]^ which prompted our hypothesis that LIMK1 and CDK5 cooperate to phosphorylate β‐catenin, enhance its interaction with NUP93, and facilitate its nuclear translocation. Indeed, ectopic expression of wild‐type LIMK1 or CDK5, but not LIMK1‐D460A or CDK5‐K33T mutant, significantly increased the interaction between β‐catenin and NUP93 (Figure 5H,I). Moreover, mutation of β‐catenin at S191, the phosphorylating modification site, diminished its physical binding to NUP93 (Figure 5J). Taken together, these results demonstrate that LIMK1 is a novel kinase responsible for the phosphorylation and nuclear accumulation of β‐catenin, in synergy with CDK5.

### 2.7 Upregulation of LIMK1 and CDK5 Promotes Tumor Metastasis and Correlates with Poor Prognosis in ESCC

**Figure 6:**
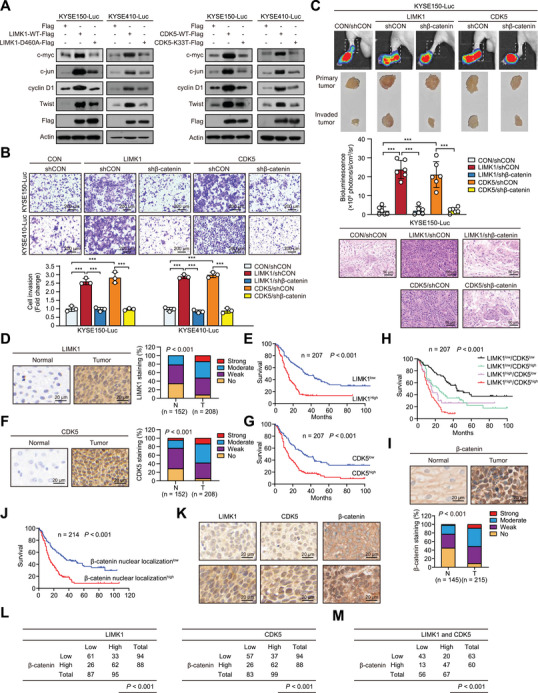
LIMK1 and CDK5 are clinically and functionally important for tumor metastasis in multiple cancer types. A) Western blot was used to detect the expression of downstream proteins in the Wnt signaling pathway in ESCC cells overexpressing LIMK1/CDK5 and their mutant type. B) Boyden chamber invasion assay showed that knockdown of β‐catenin attenuated the effect of LIMK1 or CDK5 on ESCC cell invasion. C) Bioluminescence imaging and quantitative analysis showed that β‐catenin mediated the effect of LIMK1 or CDK5 on ESCC metastasis. D) Representative images and expression patterns of LIMK1 in 208 ESCC and 152 normal tissues are shown. E) Survival analysis for 207 ESCC patients stratified by tumor LIMK1 expression levels. F) Representative images and expression patterns of CDK5 in ESCC tissue microarray are shown. G) Survival analysis for 207 ESCC patients stratified by tumor CDK5 expression levels. H) Survival analysis for 207 ESCC patients stratified by tumor LIMK1 and CDK5 expression levels. I) Representative images and nuclear localization patterns of β‐catenin in 215 ESCC and 145 normal tissues are shown. J) Survival analysis for 214 ESCC patients stratified by tumor β‐catenin nuclear localization levels. K) Representative images of β‐catenin nuclear localization at different expression levels of LIMK1 and CDK5 in ESCC tissue microarray. L, M) Association between LIMK1 and/or CDK5 with β‐catenin nuclear localization in ESCC tissues.

Nuclear translocation of β‐catenin is crucial for its role in initiating gene transcription. As expected, ectopic expression of LIMK1 or CDK5, but not LIMK1‐D460A or CDK5‐K33T mutants, significantly increased the expression of β‐catenin target genes, including c‐myc, c‐jun, cyclin D1 and twist in ESCC cells (**Figure** **6**A). Converse results were obtained by genetically manipulating the expression of LIMK1 and CDK5 via siRNA (Figure S7A, Supporting Information). In addition, knockdown of β‐catenin abolished the promoting effect of LIMK1/CDK5 (Figure S7B, Supporting Information). The Boyden chamber invasion assay showed that LIMK1 or CDK5 overexpression enhanced ESCC cell invasion, while knockdown or knockout of β‐catenin by shRNA or sgRNA significantly abrogated these effects (Figure 6B; Figure S7C, Supporting Information). In addition, a footpad lymph node metastasis model was established, and significantly increased metastasis was observed in the lymph nodes of mice injected with LIMK1 or CDK5 overexpressing ESCC cells, compared to mice injected with control cells. The enhanced metastasis was abolished when β‐catenin expression was inhibited (Figure 6C). These results collectively suggest that β‐catenin not only serves as the substrate of LIMK1 and CDK5 but also mediates their effect on tumor metastasis.

The clinical relevance of LIMK1 and CDK5 in ESCC remains unclear. By analyzing an ESCC tissue microarray (TMA) consisting of 208 tumor tissues and 152 matched adjacent normal tissues, high expression of LIMK1 (52.40%, 109/208) was observed in human ESCC tissues compared with paired normal tissues (21.71%, 33/152) (*P* < 0.001, Figure 6D). Kaplan‐Meier survival analysis indicated that patients with high LIMK1 expression had markedly shorter survival times (median survival time = 14.0 months) than patients with low LIMK1 expression (median survival time = 33.0 months) (*P* < 0.001; Figure 6E). Similarly, high expression of CDK5 (58.17%, 121/208) was observed in human ESCC tissues compared with paired normal tissues (23.68%, 36/152) (*P* < 0.001; Figure 6F). Patients with high CDK5 expression had markedly shorter survival times (median survival time = 15.0 months) than patients with low CDK5 expression (median survival time = 32.0 months) (*P* < 0.001; Figure 6G). High LIMK1 or CDK5 expression was significantly associated with pathological N stage and tumor grade (*P* < 0.05, **Tables** **1** and **2**). Furthermore, a positive correlation between LIMK1 and CDK5 expression was observed (P < 0.01, Figure S8, Supporting Information). ESCC patients with simultaneously higher expression of LIMK1 and CDK5 had significantly worse prognosis than patients with lower expression of both or one of them (*P* < 0.001, Figure 6H). To further analyze β‐catenin activation status in ESCC patients with high LIMK1 and CDK5 expression, tissue microarrays containing 215 tumor tissues and 145 matched adjacent normal tissues were stained for β‐catenin and analyzed for nuclear localization. The results showed significantly increased nuclear localization of β‐catenin in tumor samples (51.63%, 111/215) compared with paired normal tissues (22.76%, 33/145) (P < 0.001, Figure 6I). Meanwhile, patients with nuclear β‐catenin accumulation had a significantly shorter survival time (median survival time = 14.0 months) than patients with low nuclear localization of β‐catenin (median survival time = 35.0 months) (P < 0.001; Figure 6J). Moreover, nuclear localization of β‐catenin was significantly associated with pathological N stage and tumor grade (P < 0.05, **Table** **3**). A strong positive correlation was observed between nuclear localization of β‐catenin and high LIMK1 and/or CDK5 expression (Figure 6K–M). Collectively, these data suggest that upregulation of LIMK1 and CDK5 activates β‐catenin, promoting tumor metastasis and correlating with poor prognosis in ESCC.

**Table 1:** Correlation between LIMK1 expression levels and clinicopathological parameters in 208 patients with esophageal cancer.

Variable	n	Low LIMK1	High LIMK1	*P* value
Age (years)				
≤55	34	14	20	
>55	171	83	88	0.432
				
Gender				
Female	46	27	19	
Male	162	71	91	0.075
				
T stage				
1/2	31	17	14	
3/4	174	78	96	0.3
				
N stage				
N0	88	52	36	
N1/N2/N3	116	42	74	0.001**
				
TNM stage				
I & II	152	78	74	
III & IV	56	20	36	0.046*

Abbreviations: T, tumor invasion depth; N, lymph node involvement; TNM, tumor node metastasis.

*P* values < 0.05 were considered statistically significant and the levels are denoted as ^∗^, *P*  < 0.05; ^∗∗^, *P*  < 0.01; ^∗∗∗^, and *P*  < 0.001.

**Table 2:** Correlation between CDK5 expression levels and clinicopathological parameters in 208 patients with esophageal cancer.

Variable	n	Low CDK5	High CDK5	*P* value
Age (years)				
≤55	34	16	18	
>55	171	70	101	0.51
				
Gender				
Female	46	18	28	
Male	162	69	93	0.67
				
T stage				
1/2	31	13	18	
3/4	174	71	103	1
				
N stage				
N0	88	44	44	
N1/N2/N3	116	42	74	0.048*
				
TNM stage				
I & II	152	73	79	
III & IV	56	14	42	0.0028**

Abbreviations: T, tumor invasion depth; N, lymph node involvement; TNM, tumor node metastasis.

*P* values < 0.05 were considered statistically significant and the levels are denoted as ^∗^, *P*  < 0.05; ^∗∗^, *P*  < 0.01; ^∗∗∗^, and *P*  < 0.001.

**Table 3:** Correlation between β‐catenin nuclear localization and clinicopathological parameters in 215 patients with esophageal cancer.

Variable	n	Low β‐catenin nuclear localization	High β‐catenin nuclear localization	*P* value
Age (years)				
≤55	32	12	20	
>55	179	93	86	0.132
				
Gender				
Female	47	27	20	
Male	168	78	90	0.182
				
T stage				
1/2	35	22	13	
3/4	174	80	94	0.0683
				
N stage				
N0	92	54	38	
N1/N2/N3	120	49	71	0.00991**
				
TNM stage				
I & II	164	88	76	
III & IV	51	17	34	0.0112*

Abbreviations: T, tumor invasion depth; N, lymph node involvement; TNM, tumor node metastasis.

*P* values < 0.05 were considered statistically significant and the levels are denoted as ^∗^, *P*  < 0.05; ^∗∗^, *P*  < 0.01; ^∗∗∗^, and *P*  < 0.001.

To study the broad applicability of LIMK1 and CDK5 as cancer biomarkers and targets, their expression and biological functions were verified in multiple cancer types. Interestingly, the TCGA database showed that LIMK1 and CDK5 mRNA expression was not only significantly upregulated in ESCC but also in esophageal adenocarcinoma (EAC), another pathological subtype of esophageal cancer prevalent in Western counties. In addition, consistent results were obtained in gastric cancer and lung cancer (Figure S9A,B, Supporting Information). Western blot analysis of human gastric cancer and lung cancer specimens confirmed that LIMK1/CDK5 protein expression was significantly increased in primary tumors and further enhanced in matched metastatic tumor tissues compared to adjacent normal tissues (Figure S9C, Supporting Information). Moreover, gastric cancer and lung cancer patients with high LIMK1 expression had markedly shorter survival times according to the TCGA database (Figure S9D, Supporting Information). The Boyden chamber assay showed that LIMK1/CDK5 significantly promoted the invasion of EAC, gastric cancer, and lung cancer cells in a phosphorylation‐dependent manner (Figure S9E,F, Supporting Information). Furthermore, LIMK1/CDK5 also increased the phosphorylation of β‐catenin in both gastric cancer and lung cancer cells (Figure S9G,H, Supporting Information). The above findings indicate that LIMK1 and CDK5 are potential functional targets and biomarkers in ESCC, and may also apply to multiple cancer types.

### 2.8 The Combination Therapy Targeting LIMK1 and CDK5 Significantly Inhibits Tumor Metastasis

**Figure 7:**
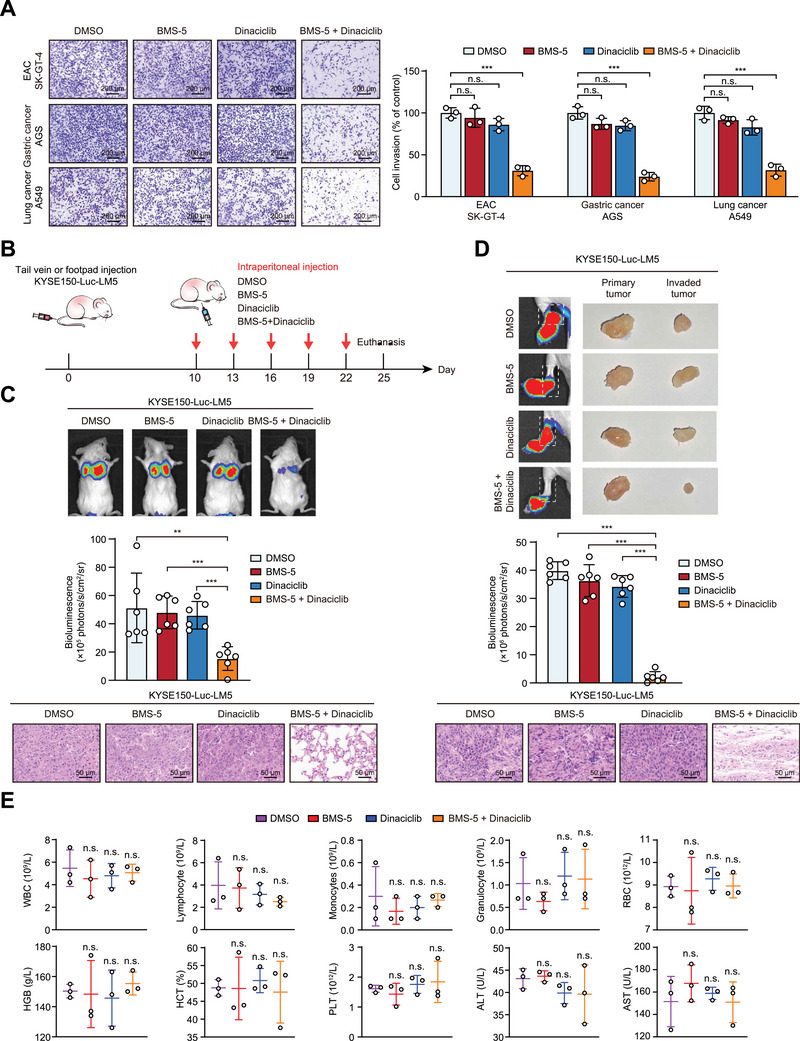
The combination therapy targeting LIMK1 and CDK5 significantly inhibits ESCC metastasis. A) Transwell assays were performed to determine the invasive abilities of EAC, gastric cancer, and lung cancer cells when treated with BMS‐5 and/or Dinaciclib. B) The flow diagram shows the lung metastasis model, lymph node metastasis model in mice, and the treatment plan. C) Bioluminescence imaging and quantification of lung metastasis in mice intravenously injected with ESCC cells and treated with BMS‐5 and/or Dinaciclib. Hematoxylin‐eosin (H&E) staining shows lung metastasis as indicated. D) The lymph node metastasis model was established and the therapeutic effects of BMS‐5 and/or Dinaciclib were monitored by Bioluminescence imaging. E) Key indicators of drug toxicity were detected in mice treated with indicated inhibitor or DMSO.

To evaluate the universal applicability of combining LIMK1 and CDK5 kinase inhibitors in tumor therapy, the Boyden chamber assay was performed in EAC, gastric cancer, and lung cancer cells. The results showed that the combination of both inhibitors significantly suppressed EAC, gastric cancer, and lung cancer cell invasion (**Figure** **7**A; Figure S10A, Supporting Information). The potential of this combination therapy was also evaluated in a preclinical setting (Figure 7B). First, KYSE150‐Luc‐LM5 cells were injected into mice to establish a lung metastasis model and drugs were intraperitoneally injected into mice every three days. Interestingly, a single kinase inhibitor did not significantly suppress metastasis, while the combination therapy led to a remarkable reduction in lung metastasis with an inhibitory rate of 69.9% (Figure 7C). Moreover, the lymph node metastasis model was constructed, and consistently, both kinase inhibitors significantly suppressed swollen inguinal lymph node metastases with an inhibitory rate of 94.7% (Figure 7D). The bioluminescence imaging data was also evidenced by histological examination of the lung and metastatic lymph nodes (Figure 7C,D). Furthermore, to assess the systemic toxicity of BMS‐5 and/or Dinaciclib treatments, morphological changes in key organs and analyzing a spectrum of hematological parameters were examined. The results showed no significant alterations between treated and untreated groups (Figure 7E; Figure S10B, Supporting Information). In addition, CCK‐8 assay results revealed no significant impact on normal esophageal cell proliferation (Figure S10C, Supporting Information), further suggesting the safety of this treatment strategy. Based on these findings, we proposed a combined therapeutic strategy of simultaneously targeting LIMK1 and CDK5 for the treatment of tumor metastasis, especially in ESCC.

## 3 Discussion

**Figure 8:**
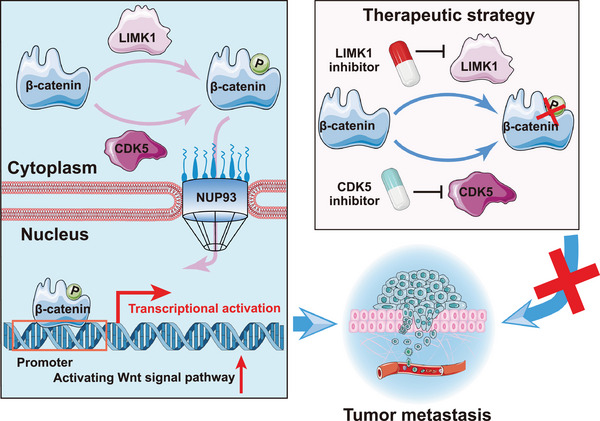
Schematic model.

In this study, we present a comprehensive kinase‐substrate map of metastatic ESCC and generate a high‐quality multi‐omics data resource. We emphasize the important role of phosphorylation in the Wnt/β‐catenin pathway in ESCC metastasis. We provide the first evidence that LIMK1 is a novel kinase that, in synergy with CDK5, is responsible for phosphorylating β‐catenin at S191, leading to β‐catenin nuclear translocation and ultimately promoting ESCC metastasis. These findings also proposed that the treatment strategy of simultaneously targeting LIMK1 and CDK5 may hold promising prospects for precision treatment of metastatic human cancer (**Figure** **8**).

First, we report for the first time that LIMK1 functions as a kinase for β‐catenin phosphorylation, regulating its unclear accumulation and activation. Beyond RNA/protein abundance, our analysis included protein activity inferred from phosphoproteomics data, identifying additional key proteins that could not be identified from global transcriptome/proteomics. Based on this, we found that the Wnt/β‐catenin signaling pathway was the most highly regulated by phosphorylation in metastatic ESCC. The phosphorylation and nuclear accumulation of β‐catenin is recognized as a critical feature for the activation of the Wnt/β‐catenin pathway.^[^ ^11^ ^]^ β‐catenin lacks a nuclear localization signal, and accumulating evidence suggests that it employs non‐classical mechanisms for nuclear entry.^[^ ^27^ ^]^ However, the complex regulatory network of β‐catenin phosphorylation remains largely unknown, especially the kinases that regulate its phosphorylation. How phosphorylation precisely regulates β‐catenin nuclear translocation also warrants investigation. In this study, we prove that LIMK1 directly binds to the ARM domain and phosphorylates the S191 site of β‐catenin. This phosphorylation modulates the interaction between β‐catenin and NUP93 to facilitate nuclear accumulation and activate downstream pathways. Our current work enriches our comprehension of β‐catenin nuclear import and expands the kinase regulatory network of Wnt/β‐catenin signaling.

Second, our current work demonstrates the essential role of LIMK1 and CDK5 in tumor metastasis. Metastasis is the hallmark of cancer and is responsible for ≈90% of cancer‐related deaths, but it remains poorly understood. Before developing distant metastases, ESCC often forms metastases in adjacent lymph nodes.^[^ ^28^ ^]^ Therefore, using lymph node metastatic tissues to find effective biomarkers for discerning cases with lymph node metastasis cases from early‐stage patients is an efficient strategy. However, there has been a lack of systematic and multilevel analysis of metastatic lymph nodes in esophageal cancer to date. In this study, we integrated multi‐omics analysis with CRISPR/Cas9‐based functional screening to systematically identify key kinases that drive ESCC metastasis. We found that LIMK1/CDK5 is highly expressed and hyperactivated in tumors, especially in lymph node metastatic tissues. High expression of LIMK1 and CDK5 was significantly correlated with poor prognosis of ESCC. In addition, we demonstrated that LIMK1 collaborates with CDK5 to activate β‐catenin signaling and promote ESCC metastasis in vitro and in vivo. The clinical and functional significance of the LIMK1/CDK5‐Wnt/β‐catenin axis was also verified in esophageal adenocarcinoma, gastric cancer, and lung cancer. These results highlight the potential and the broad applicability of LIMK1 and CDK5 as prognostic biomarkers in human cancer, providing a rationale for simultaneously targeting these kinases as a cancer therapeutic strategy.

Third, our findings provide a novel treatment strategy to impede ESCC metastasis by inhibiting the phosphorylation of β‐catenin. Esophageal cancer ranks as the sixth most common cause of cancer‐related death worldwide and predominates in Eastern Asia and Africa.^[^ ^29^ ^]^ It is characterized by asymptomatic early stages, often leading to diagnosis at locally advanced or metastatic stages, resulting in a five‐year survival rate of 22%. There is an urgent need to explore the mechanisms of metastasis and to identify effective biomarkers for diagnosis, prognosis, and treatment of metastatic cancer.^[^ ^30^ ^]^ The Wnt/β‐catenin signaling pathway is an attractive diagnostic biomarker and therapeutic target. Nuclear β‐catenin localization in immunohistochemistry can stratify patients for targeted therapies. However, targeting β‐catenin remains a challenging strategy, as it is difficult to directly target β‐catenin using small‐molecule compounds.^[^ ^17^ ^]^ Although various Wnt/β‐catenin inhibitors have been developed, they have demonstrated limited antitumor activity and significant cytotoxicity in clinical trials.^[^ ^31^, ^32^ ^]^ This has led to the absence of approved drugs targeting β‐catenin and a lack of successful clinical applications of these inhibitors. Therefore, targeting upstream regulatory kinases of the Wnt/β‐catenin pathway may represent a more promising therapeutic strategy. Over the past years, a vast number of kinases have been reported to be strongly associated with cancer, positioning protein kinase inhibition as an emerging therapeutic strategy.^[^ ^33^, ^34^ ^]^ Among these, CDK5 has been highlighted as a valuable target in cancer treatment. From pan‐CDK inhibitors to pathway‐specific blocking peptides, the repertoire of CDK5‐targeting drugs is increasing.^[^ ^35^ ^]^ However, till now, the outcomes of clinical trials with CDK5 inhibitors have been unsatisfactory due to cytotoxicity or modest efficacy.^[^ ^36^, ^37^ ^]^ Combination therapy, which works in a synergistic or additive manner, can increase treatment efficacy while lowering the therapeutic dosage of one agent, thereby reducing toxic effects. Indeed, combining CDK5 inhibitors with other agents, such as tilorone, presents a promising method to boost efficacy in prostate cancer.^[^ ^38^ ^]^ Previous combination therapies targeting β‐catenin resulted in its degradation,^[^ ^39^ ^]^ which was frequently associated with cytotoxicity, thus limiting their clinical applicability. Therefore, selectively targeting multiple kinases in the Wnt/β‐catenin pathway to reduce abnormal nuclear accumulation of β‐catenin is a common strategy to construct more effective and safer treatments, while leaving fewer redundant escape routes for the tumor cell. Inspiring, we further extended the regulatory network of β‐catenin function and elucidated the intrinsic mechanism by which LIMK1 and CDK5 perform similar functions, introducing novel insights for targeted therapy of cancer treatment using CDK5 in combination with another agent. In this study, combination therapy with LIMK1 and CDK5 inhibitors exhibited a significant therapeutic effect on ESCC metastasis in a preclinical setting, as well as in EAC, gastric cancer, and lung cancer metastasis. Our findings provide a promising strategy for the treatment of cancer metastasis.

## 4 Experimental Section

### Clinical Sample Acquisition

All human specimens used in this study were collected from newly diagnosed patients who underwent resection surgery at Shanghai Chest Hospital, Shanghai Jiao Tong University, and the First Hospital of China Medical University. Written informed consent was obtained from each patient, and all procedures were conducted in accordance with routine clinical practice. Each tissue specimen was collected within 30 min after surgical resection and immediately transferred to liquid nitrogen for rapid freezing. They were subsequently stored at −80 °C until used for RNA sequencing, proteomics, phosphoproteomics, and Western blot analysis.

### Protein Extraction and Digestion

Protein extraction and digestion were performed as previously described.^[^ ^40^ ^]^ In brief, all tissues were ground into powder and then added to a lysis buffer, followed by sonication at 20% amplitude for a total working time of 3 min, with 3s on and 3s off, to lyse the tissues while carefully maintaining a low temperature. For phosphoproteomics experiments, a 1% phosphatase inhibitor (PhosSTOP Roche) was needed for the lysis buffer. After centrifugal supernatant on collection, concentration, and determination, and an equivalent amount of protein was used for pancreatic enzyme digestion. Finally, the peptides were desalted and dried for the following experiment.

### Bio‐Material‐Based Phosphopeptide Enrichment

Peptide mixtures were initially incubated with immobilized metal affinity chromatography (IMAC) microspheres suspended with vibration in a loading buffer (50% acetonitrile/0.5% acetic acid) for 30 min. To remove non‐specifically adhered peptides, the IMAC microspheres underwent a thorough washing procedure. They were first rinsed with a solution consisting of 50% acetonitrile and 0.5% acetic acid, followed by a subsequent wash with a mixture of 30% acetonitrile and 0.1% trifluoroacetic acid. To elute the enriched phosphopeptides, an elution buffer containing 10% NH_4_OH was added and the peptides were eluted with vibration. The resulting supernatant, abundant in phosphopeptides, was collected and subsequently subjected to lyophilization in preparation for the following experiment.

### Peptides Fractionation

To minimize the probability of peptides co‐eluting and co‐fragmenting in these highly intricate samples, peptides were fractionated using high‐performance liquid chromatography (HPLC). The separation of peptides was achieved through a nonlinear increasing concentration of solvents, from solvent buffer A (2% acetonitrile in 10 mm ammonium bicarbonate with pH equal to 10) to solvent buffer B (60% acetonitrile in 10 mm ammonium bicarbonate with pH equal to 10). The eluate was auto‐collected at 1‐min intervals, resulting in 80 fractions. Subsequently, these fractions were merged into nine fractions and dried. Each fraction was individually analyzed with LC‐MS/MS settings as described below.

### Proteomic Analysis

LC‐MS/MS analysis was performed as previously described.^[^ ^40^ ^]^ In brief, 0.1% formic acid was used to dissolve 1 µg of peptides, which were subsequently analyzed using the timsTOF Pro mass spectrometry. The electrospray voltage was set at 1.7 kV, and the precursors and fragments were analyzed by the detector which was operated in parallel accumulation serial fragmentation (PASEF) mode, within a m/z range from 100 to 1700. Precursors with charge states from 0 to 5 were selected for fragmentation and ten PASEF‐MS/MS scans were acquired in each cycle. A dynamic exclusion setting of 30 s was implemented to prevent the reselection of recently analyzed peptides.

### Phosphoproteomic Analysis

The tryptic peptides were reconstituted in solvent buffer C (2% acetonitrile with 0.1% formic acid in water). These peptides were subsequently directly introduced onto a custom‐made reversed‐phase analytical column (25 centimeters in length, 75 micrometers in inner diameter). The peptides were effectively separated by employing a gradient elution method: commencing at 5% solvent buffer D (90% acetonitrile with 0.1% formic acid), the gradient increased to 25% over a duration of 60 min. This was followed by a shift to 35% over 22 min and a rapid ascent to 80% in just 4 min then keeping at 80% for the last 4 min. The final 4 min maintained a consistent flow rate of 450 nL min^−1^. All of this was conducted on an EASY‐nLC 1200 UHPLC system at a constant flow rate of 450 nL min^−1^ and the products of separation were analyzed with mass spectrum (Q Exactive HF‐X). An electrospray voltage of 2.0 kV was precisely applied. The full mass spectrum scan was performed with a resolution of 60000, covering a scan range of 350–1600 m/z. Up to 20 of the most abundant precursors were chosen for subsequent MS/MS analyses with a dynamic exclusion period of 30 s. To ensure precise data collection, the Automatic Gain Control (AGC) target was established at 1E5, with an intensity threshold of 3.3E4, and a maximum injection time of 60 ms was diligently enforced. The phosphorylation sites from the same phosphoproteins were collapsed by calculating the median ratio. Differential expression analysis between N, T, and LN was performed using the limma R package (version 3.54.0).

### Database Searching of MS Data

All mass spectrometric data were subjected to analysis using the MaxQuant (v.1.6.15.0). To avoid calculating the false discovery rate (FDR) from being inflated by random matches, tandem mass spectra were searched against the human Swiss‐Prot database (20422 entries), which was combined with a reverse decoy database. Additionally, common contaminating libraries were included to account for contaminating proteins. Trypsin/P was designated as the cleavage enzyme, allowing for a maximum of 2 missed cleavages. A mass tolerance of 20 ppm was applied to precursor ions in the initial search, while a tighter tolerance of 5 ppm was used in the main search. For fragment ions, the mass tolerance was precisely set at 0.02 Da. Carbamidomethyl modification on Cysteine residues was searched as a fixed modification. Acetylation on protein N‐terminal, phosphorylation on serine, threonine, and tyrosine, and oxidation on methionine were specified as variable modifications. In order to obtain high‐quality analysis results, further data filtering was applied, setting the FDR at 1% for spectrum, peptide, and protein levels. Additionally, identified proteins were required to contain at least one unique peptide.

### Proteomics and Phosphoproteomics Data Normalization

Global protein and phosphosite abundance from 60 samples were measured by label‐free proteomic quantification techniques. The LFQ intensities (I) of the proteins and signal intensity values (I) of the modified peptides in each sample were centered to derive relative quantitative values (R). The formula for calculation is as follows, where “i” represents the sample and “j” represents the protein:

(1) $$R_{ij}=I_{ij}/Mean\left(I_{j}\right)$$

The relative quantitative value of the modification site was normalized by dividing it by the relative quantitative value of the corresponding protein, effectively removing the impact of protein expression on the modified expression.

### Differential Expression Analysis

For samples to be compared, the mean relative quantitative values of each protein across multiple replicates were utilized to determine the fold change (FC). For instance, to compute the FC of proteins between sample groups A and B, the formula is as follows: where “R” represents the relative quantitative value of the protein, “i” represents the sample, and “k” represents the protein.

(2) $$FC_{A/B,k}=\;\mathrm{Mean}\left(R_{\mathrm{ik}},\;i\in \;A\right)/\mathrm{Mean}\left(R_{\mathrm{ik}},\;i\in \;B\right)$$

To assess the significance of differences, the relative quantitative values of each protein in the comparison group samples underwent a t‐test, with the resulting *P* value indicating significance, typically set at a default threshold of *P* < 0.05. Before conducting the test, the relative quantitative values were log_2_ transformation to satisfy the t‐test normal distribution assumption. The transformation formula is as follows:

(3) $$P_{k}=T.test\left(log_{2}\left(R_{ik},\;i\in \;A\right),log_{2}\left(R_{ik},\;i\in \;B\right)\right)$$

### RNA‐Seq and Quantification

After excluding samples that failed quality control, RNA was extracted from 51 samples, including N, T, and LN, for RNA‐Seq. The concentration and RNA integrity were assessed, and only those meeting quality control standards were used for library preparation. Sequencing libraries were generated using the MGIEasy RNA Library Prep kit (MGI) according to the manufacturer's instructions, with index codes added to link sequences to each sample. Eventually, circularized DNA was sequenced with the MGISEQ‐2000 system. After the removal of adaptors and low‐quality reads, the cleaned RNA‐seq data were aligned to the human reference genome (GRCh38.p13 assembly) using Bowtie2. The mapped reads were assembled into genes using RSEM (version 1.3.1) to quantify gene expression levels in transcripts per million (TPM). For the analysis of differential gene expression, the DESeq2 R package (version 1.42.0) was utilized to compare read counts per gene across samples in the RNA‐seq dataset.

### Survival Analysis

Progression‐free survival (PFS) and overall survival (OS) were conducted using the log‐rank test. Kaplan–Meier curves were created to visualize survival data. The Cox proportional hazards model assessed the hazard ratio (HR) and statistical significance for each gene.

### Hallmark Gene Set Analysis

The hallmark gene sets, comprising 50 representative pathways encompassing well‐defined genes involved in developmental, immune, and signaling pathways, were obtained from the Molecular Signatures Database (MsigDB).^[^ ^41^ ^]^ The integrated abundance of proteins associated with these hallmarks in each sample using the GSVA R package (version 1.50.0) was calculated, utilizing a normalized phosphoprotein expression matrix. Differences in GSVA scores for pathways among the three tissues were assessed using the Wilcoxon signed‐rank test.

The Hallmark Wnt/β‐catenin signaling and epithelial‐mesenchymal transition gene sets were utilized to calculate the average expression of each sample's gene sets. Differences in the average expression of these gene sets among the three tissues were evaluated using the Wilcoxon signed‐rank test.

### Kinase Activity Prediction

Kinase‐substrate pairs were collected from dbPTM (https://awi.cuhk.edu.cn/dbPTM) and benchmark data collected from predictive models of kinase‐substrate regulatory relationships, including GPS5.0,^[^ ^42^ ^]^ iGPS^[^ ^43^ ^]^ and musiteDeep,^[^ ^44^ ^]^ creating GMT files. The single‐sample gene set enrichment analysis (ssGSEA) was employed to calculate kinase activities based on the expression levels of all identified phosphosites. Additionally, kinase activities were categorized into four categories according to the classification criteria of tissue‐associated phosphoproteins. Differences among the three tissues were evaluated using Student's t‐test. Log_2_FC was calculated as the median expression difference for the same proteins across different samples.

### Analysis of Phospho‐Regulatory Network

Human protein‐protein interactions (PPIs) from the STRING database was utilized.^[^ ^45^ ^]^ Kinases, substrates, and proteins from the Wnt signaling pathway, Insulin signaling pathway, and epithelial‐mesenchymal transition showing sequential elevation expression across N, T, and LN, and specific highest expression in LN, were mapped to construct the PPI network. Pair‐wised Spearman correlation coefficients were used to assess correlations between kinases and their substrates, as well as between substrates and protein in oncogenic signaling pathways (kinase‐substrate r ≥ 0.3, *P* < 0.05; substrate‐oncoprotein |r| ≥ 0.5, *P* < 0.05). The resulting network was visualized using Cytoscape (version 3.10.2).^[^ ^46^ ^]^

### Druggable Kinases and Cancer‐Associated Substrates

Correlations for all known kinase‐substrate pairs and those predicted by GPS5.0^[^ ^42^ ^]^ (medium threshold) were calculated, using Pearson correlation coefficients based on kinase activity and phosphosite expression in N, T, and LN for each patient. A kinase‐substrate pair correlation was deemed high confidence if it was greater than 0.5 in 75% of patients. FDA‐approved and potential drug targets were compiled from the Human Protein Atlas^[^ ^47^ ^]^ (https://www.proteinatlas.org), resulting in 2079 defined genes as drug targets. High‐confidence kinase‐substrate pairs were visualized based on two criteria: 1) the kinase is a drug target and the substrate is a cancer‐associated gene; 2) the protein expression of both the kinase and the substrate in the pair is incrementally elevated in N, T, and LN or uniquely highly expressed in LN.

### Kinase Recognition Motif

CDK5 and LIMK1 substrates were extracted separately, with 4 amino acids upstream and downstream of the substrate phosphorylation site forming a 9‐amino‐acid peptide. Additionally, 1000 non‐phosphorylated substrate peptides of CDK5 and LIMK1 were randomly selected using the same method. Peptide lengths were verified using Seqkit,^[^ ^48^ ^]^ followed by motif identification for CDK5 and LIMK1 recognition using MEME. Finally, FIMO scanned the CTNNB1 sequence for matches to each CDK5 and LIMK1 motif individually.

### Cell Culture and Drugs

Human ESCC cell lines KYSE150 and KYSE410 were purchased from Deutsche Sammlung von Mikroorganismen und Zellkulturen (DSMZ, Braunschweig, Germany). The NE1 line was gifts from Prof. George Tsao and Dr. Annie Cheung. The human EAC cell lines SK‐GT‐4, FLO‐1, and the human gastric cancer cell lines AGS, MKN28 were obtained from the Institute of Biochemistry and Cell Biology at the Chinese Academy of Sciences (Shanghai, China). The human lung cancer cell lines A549, and H1299 were purchased from ATCC (Rockville, MD, USA). Through a series of in vivo and in vitro selection procedures, highly metastatic and invasive sublines of ESCC, namely KYSE150‐Luc‐LM5 and KYSE410‐Luc‐I3, were established, as detailed in previous descriptions.^[^ ^49^ ^]^ All cells were cultured in RPMI‐1640 or DMEM cell culture medium supplemented with 10% FBS at 37 °C in a 5% CO_2_ atmosphere. To prevent any potential cross‐contamination, short tandem repeat profiling was employed for all cell lines and tested negative for mycoplasma. BMS‐5 and Dinaciclib were purchased from Selleck Chemicals (Houston, TX, USA).

### Genome‐Wide CRISPR/Cas9 Screening

The human CRISPR knockout pooled library (GeCKO v2, Addgene plasmid #1000000048; Addgene), encompassing 123411 unique sgRNAs targeting 19050 annotated protein‐coding human genes (6 sgRNA per gene) and 2000 control sgRNAs, was a gift from Feng Zhang (Massachusetts Institute of Technology, Cambridge, MA). Genome‐wide CRISPR/Cas9 screening was performed as previously described.^[^ ^50^ ^]^ In brief, cells were transduced with the pooled lentiviral GeCKO v2 library. After puromycin selection, the cells were divided into a control group which serves as input DNA, and an experimental group. For the in vitro screening, the experimental group was subjected to three successive selections within a Matrigel‐coated Boyden chamber, with subsequent cell harvesting for downstream sequencing. For the in vivo screening, the cells of the experimental group were injected intravenously via the tail vein into NOD‐Prkdc^em26Cd52^Il2rg^em26Cd22^ (NCG) mice. After the occurrence of mouse lung metastasis, the metastatic cells were collected for sequencing.

### Plasmids, Transfection, Infection, and Gene Knockdown or Expression by CRISPR/Cas9 Genome Editing

All wild‐type overexpression plasmids, small interfering RNAs(siRNA), and the short hairpin RNAs (shRNA) against β‐catenin were obtained from TranSheep Bio (Shanghai, China). Point mutations and truncation mutations were generated from the corresponding wild‐type plasmids via PCR amplification and then cloned into the corresponding empty vector using a pEASY‐Basic Seamless Cloning and Assembly Kit (TransGen Biotech Co., LTD) according to the manufacturer's instructions. All plasmids were ultimately confirmed by full‐length DNA sequencing. Plasmids and siRNA were transiently transfected in corresponding cells using Lipofectamine 3000 reagent (Invitrogen) according to the manufacturer's protocol. For the establishment of stable cell lines, LIMK1, CDK5 overexpression, or shβ‐catenin plasmids were transfected into HEK293T cells together with the 3rd generation lentiviral packaging plasmids (Addgene #12251, #12253 and #12259). The cell culture medium was collected 48 h post‐infection. After centrifugation and filtration, the viral supernatant was obtained. Subsequently, the ESCC cells were infected with this viral supernatant. Appropriate antibiotics were selected based on the resistance genes present on the plasmid to screen the infected cells. The protein levels were assessed using western blot assay. Details regarding the primers, siRNA, and shRNA utilized in this study can be found in Table S5 (Supporting Information).

### Tissue Microarray and Immunohistochemistry (IHC) Analyses

ESCC tissue microarrays (Shanghai Outdo Biotech, Shanghai, China) were used to analyze the expression of LIMK1, CDK5, and β‐catenin nuclear localization. IHC was performed as previously described.^[^ ^51^ ^]^ In brief, microarrays were de‐paraffinized in xylene and rehydrated in a descending ethanol series (100%, 95%, 90%, 85%, 80%, 70% of ethanol). After antigen retrieval using 0.01 mol L^−1^ citrate buffer at pH 6.0, microarrays were quenched for endogenous peroxidase activity, blocked, and incubated with corresponding primary antibodies overnight at 4 °C. After incubation with a secondary antibody, 3,3′‐Diaminobenzidine (DAB) solution was used as the substrate to visualize the immunostaining and counterstained with hematoxylin. The staining intensity of the tissue microarray was rated using this scale: 0 for negative staining, 1 for weak staining, 2 for moderate staining, and 3 for strong staining. Samples with a score of 0 or 1 were categorized as having low expression, whereas those with a score of 2 or 3 were categorized as having high expression.

### Western Blot

Western blot analysis was conducted following established protocols.^[^ ^52^ ^]^ In brief, proteins were separated by SDS‐PAGE, transferred to the PVDF membrane, and blocked with 5% milk for 90 min. The PVDF membranes were then incubated with primary antibodies, followed by incubation with secondary antibodies. The sources and dilutions of the primary antibodies were as follows: pan‐specific antibodies targeting serine phosphorylation modifications (Santa Cruz Biotechnology), as well as LIMK1, Fibronectin, VEGF, N‐cadherin, CDK5, β‐Actin (Santa Cruz Biotechnology); c‐myc (PTM Biolab Co., Ltd), β‐catenin, Lamin B, GAPDH, MYC‐tag, GST‐tag, HA‐tag, c‐jun, cyclin D1, Twist, p53 (Proteintech); E‐cadherin (BD Biosciences), and Flag (Sigma–Aldrich) antibodies. The antibodies are listed in Table S6 (Supporting Information).

### Cytoplasmic and Nuclear Protein Isolation

Cells were incubated with extraction buffer (10 mm Tris pH 7.6, 10 mm KCl, 5 mm MgCl_2_, and protease inhibitor cocktail (Roche)) for 10 min at 4 °C. Then, extraction buffer with 0.6% Triton X‐100 was added and incubated for 20 min on ice. To separate the nucleus factions, a nuclear isolation buffer (extraction buffer with 350 nm sucrose) was added and centrifuged at 600 g for 10 min. Following the collection of the cytoplasmic supernatant, the nuclear pellet was resuspended in ice‐cold SDS lysis buffer with a protease inhibitor cocktail. The mixture was sonicated at 4 °C for 20 min and then centrifuged at 10,000 g. The supernatant, containing the nuclear, was collected. Equal amounts of cytoplasmic and nuclear fractions were then applied to western blotting. Lamin B served as the lading control for nuclear extracts, while GAPDH was used for cytoplasmic extract.

### Protein Purification

The pGEX‐6P vector was utilized to generate plasmids pGEX‐6P‐β‐catenin, pGEX‐6P‐β‐catenin‐S191A, pGEX‐6P‐CDK5, pGEX‐6P‐LIMK1, which express wild‐type and mutant GST‐and His‐tagged fusion proteins. These plasmids were transformed into E. coli (BL21), and expression of β‐catenin and β‐catenin‐S191A‐GST protein was induced by adding 0.4 mm IPTG for 16 h at 16 °C when the bacterial culture's optical density at 600 nm reached ≈0.6. The bacteria were then lysed by sonication, and the GST‐tagged fusion proteins were purified using GSH according to the manufacturer's protocol. While His‐tagged proteins were isolated by Ni‐NTA affinity chromatography. The purification proteins were used in GST pull‐down assay and in vitro kinase assays as mentioned above.

### GST Pull‐Down Assay

GST or β‐catenin‐GST fusion protein (20 µg) was incubated with GSH beads for 2 h at 4 °C. The complexes were then resuspended in IP lysis buffer, and LIMK1/CDK5‐His‐tagged proteins (20 µg) were added. The mixture was incubated overnight at 4 °C. following washing, the bead‐bound proteins were analyzed by SDS‐PAGE and detected using Western blot.

### Co‐Immunoprecipitation (Co‐IP) and in vitro Kinase Assay

Co‐IP experiments were performed as described previously.^[^ ^53^ ^]^ Briefly, cell lysates were incubated with IgG and protein A/G Sepharose beads (Santa Cruz Biotechnology) at 4 °C for 1 h. The supernatant was mixed with the appropriate primary antibody overnight at 4 °C. This was followed by a 4‐h incubation with protein A/G Sepharose beads. The beads were thoroughly washed with IP lysis buffer, and proteins were eluted by boiling in SDS‐PAGE loading buffer followed by Western blot analysis. For the in vitro kinase assay, equal amounts of lysates from cells that overexpression CDK5(WT or K33T)‐Flag‐tag or LIMK1(WT or D460A)‐Flag‐tag, were incubated with Flag antibody overnight at 4 °C. The complexes were then purified using protein A/G Sepharose beads. The beads were equilibrated in kinase buffer (50 mm Tris pH 7.5, 150 mm NaCl, 10 mm MgCl_2_, 5 mm MnCl_2_, 1 mm DTT, 5 mm EGTA, 1 mm Na_3_O_4_V) and collected by centrifugation. Then, an in vitro kinase assay was performed in kinase buffer supplemented with 50 µm ATP at 30 °C for 30 min, with 4 µg of β‐catenin‐GST or β‐catenin‐S191A‐GST added as the substrate. Kinase reactions were analyzed by SDS‐PAGE and immunoblotting using the p‐Ser antibody.

### In Vitro Invasion Assay

In vitro invasion assay was performed as described previously.^[^ ^54^ ^]^ Briefly, different types of tumor cells were seeded in FBS‐free media in the upper chamber of Matrigel‐coated transwells with 8.0 µm pore size (Corning) and full media in the lower chamber. For inhibition of LIMK1 or CDK5, Corresponding inhibitors were added to the media in both upper and lower chambers throughout the assays. Then, the cells that migrated to the lower side of the membrane were fixed with methanol and stained with 0.2% crystal violet. Subsequently, the cells were observed under an inverted microscope.

### Footpad Lymph Node Metastasis Mouse Model

Sex was not considered as a biological variable. Six‐week‐old male BALB/c nude mice were purchased from the SPF (Beijing) Biotechnology Co., LTD. Each mouse (6 mice per group) received an injection of 1 × 10^6^ ESCC cells into the left footpad. After intraperitoneal injection of D‐luciferin (150 mg kg^−1^ in PBS) under anesthesia, the mice were imaged using an IVIS‐100 Imaging System (Caliper Life Sciences, Waltham, MA, USA). Tumors located in the footpad region were defined as “primary tumors”, while any tumors extending from the footpad to the ankle joint were classified as “invaded tumors” (local tumor invasion). All tumors were resected and fixed in 10% formaldehyde/PBS for histological analysis. All animal experiments were approved by the Experimental Animal Ethics Committee of Guangzhou Medical University and conducted in accordance with institutional guidelines.

### Experimental Lung Metastasis Model

Sex was not considered as a biological variable. Male NCG mice, aged 6–8 weeks, were maintained under standard conditions according to the institutional guidelines for animal care. The mice were randomly divided into different groups (6 mice per group). A metastasis model was established as previously described.^[^ ^55^ ^]^ Briefly, 5 × 10^5^ luciferase‐expressing ESCC cells were intravenously injected into the tail vein of mice. The mice were given 15 mg kg^−1^ BMS‐5 (prepared in 20% hydroxypropyl‐β‐cyclodextrin) or 10 mg kg^−1^ Dinaciclib (prepared in 20% hydroxypropyl‐β‐cyclodextrin) or their combination by intraperitoneal injection twice weekly. Metastasis was tracked weekly using the IVIS 100 Imaging System after intraperitoneal injection of D‐luciferin under anesthesia.

### Statistical Analysis

All in vitro experiments were repeated at least three times and statistical tests were performed using the GraphPad Prism version 8.0 (GraphPad Software, USA). The Statistical Package for Social Sciences 20.0 for Windows (SPSS, Chicago, IL) was used for the survival analysis of the Kaplan–Meier method. Data were presented as means ± standard deviation (SD). *P* values < 0.05 were considered statistically significant and the levels are denoted as ^∗^, *P*  < 0.05; ^∗∗^, *P*  < 0.01; ^∗∗∗^, and *P*  < 0.001. “n.s.” indicates non‐significant results.

### Ethics Approval

All animal experiments were approved by the Ethics Committee for Animal Experiments of Guangzhou Medical University, and the mice were cared for under standard conditions according to institutional guidelines. The human specimens were collected and approved by the Ethics Committee of Shanghai Chest Hospital, Shanghai Jiao Tong University, and the First Hospital of China Medical University. Informed consent was obtained from each participant.

### Code Availability

All analysis was performed using freely available R packages. Plots were generated with software and pipelines that have been in use in previous papers. No special‐purpose bioinformatic algorithms were used.

## Conflict of Interest

The authors declare no conflict of interest.

## Author Contributions

S.‐J.L., Z.‐R.L., Z.‐C.L., and X.‐P.L. contributed equally to this work. S.J.L. designed research studies, conducting experiments, acquiring data, analyzing data, and drafting the manuscript. Z.‐R.L. designed research studies, analyzing data, and drafting the manuscript. Z.C.L., X.Z.H., C.‐C.Z., F.Z., A.K.‐Y.L., W.D., M.‐L.H., B.L., Q.Z., G.‐L.L., J.‐B.L., Z.‐N.W. and Z.‐G.L. did critical revision of the manuscript for important intellectual content, technical and/or material support. X.‐P.L., J.‐Y.L., X.‐M.Y., Y.H., T.‐Y.X., J.‐J.X., S.‐C.P., Y.‐X.S., Y.H. and X.‐W.Z. did acquisition of data, analysis and interpretation of data. C.‐C.Z., F.Z., Z.‐X.L., W.‐W.X. and B.L. did funding acquisition, study concept and design, study supervision.

## Supporting information



Supporting Information

Supporting Information

Supporting Information

Supporting Information

Supporting Information

Supporting Information

Supporting Information

## Data Availability

The data that support the findings of this study are available from the corresponding author upon reasonable request.
