# Supplementary material for: LIMK1 as a Novel Kinase of β‐Catenin Promotes Esophageal Cancer Metastasis by Cooperating With CDK5
Source: Adv Sci (Weinh). 2025 Jun 6;12(29):e03223. doi: 10.1002/advs.202503223 (PMC12362812; doi:10.1002/advs.202503223)
Supplement: Supplementary file 1 — Supporting Information [file ADVS-12-e03223-s001.pdf]

ADVANCED SCIENCE
Open Access

Supporting Information

for *Adv. Sci.*, DOI 10.1002/advs.202503223

LIMK1 as a Novel Kinase of β-Catenin Promotes Esophageal Cancer Metastasis by Cooperating With CDK5

Shu-Jun Li, Zhuo-Ran Liang, Zhi-Chao Liu, Xue-Ping Luo, Jun-Yi Li, Xiao-Mei Yu, Xuan-Zhang Huang, Yan He, Tao-Yang Xu, Jiao-Jiao Xu, Shao-Cong Peng, Yu-Xiang Song, Yan He, Xiao-Wan Zhuang, Can-Can Zheng, Fan Zhang, Alfred King-Yin Lam, Wei Dai, Ming-Liang He, Bo Liu, Qi Zhao, Guo-Liang Lu, Jin-Bao Liu, Zhen-Ning Wang, Zhi-Gang Li*, Ze-Xian Liu*, Wen-Wen Xu* and Bin Li**

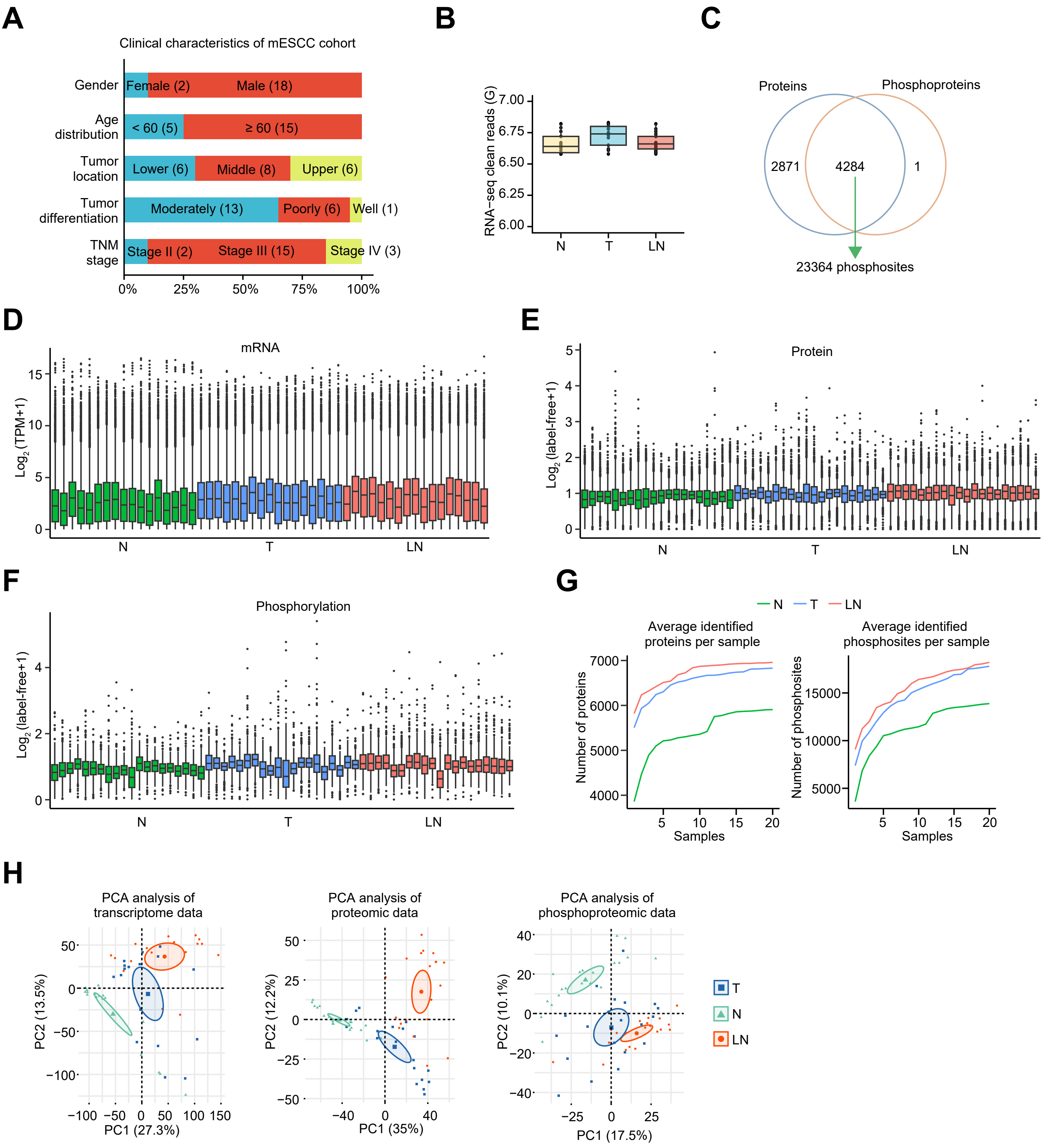

Supplementary Figure 1

Supplementary Fig. 1 Quality control of our metastatic ESCC cohort and quality assessments for MS data

(A) Summary of clinical characteristics of 20 ESCC patients with metastasis. **(B)** QC passed reads in RNA-seq for N, T and LN. **(C)** Number of overlapping proteins and their phosphorylation sites in the proteome and phosphoproteome. **(D-F)** Normalization data of RNA-Seq (D), proteomic data (E) and phosphoproteome (F) in our ESCC cohort. Red: LN; blue: T; green: N. **(G)** Cumulative number of proteins and phosphosites identified in N (green line), T (blue line) and LN (red line). **(H)** Principal-component analysis (PCA) of transcriptome, proteomic and phosphoproteome in N (green triangles), T (blue square) and LN (pink circles).

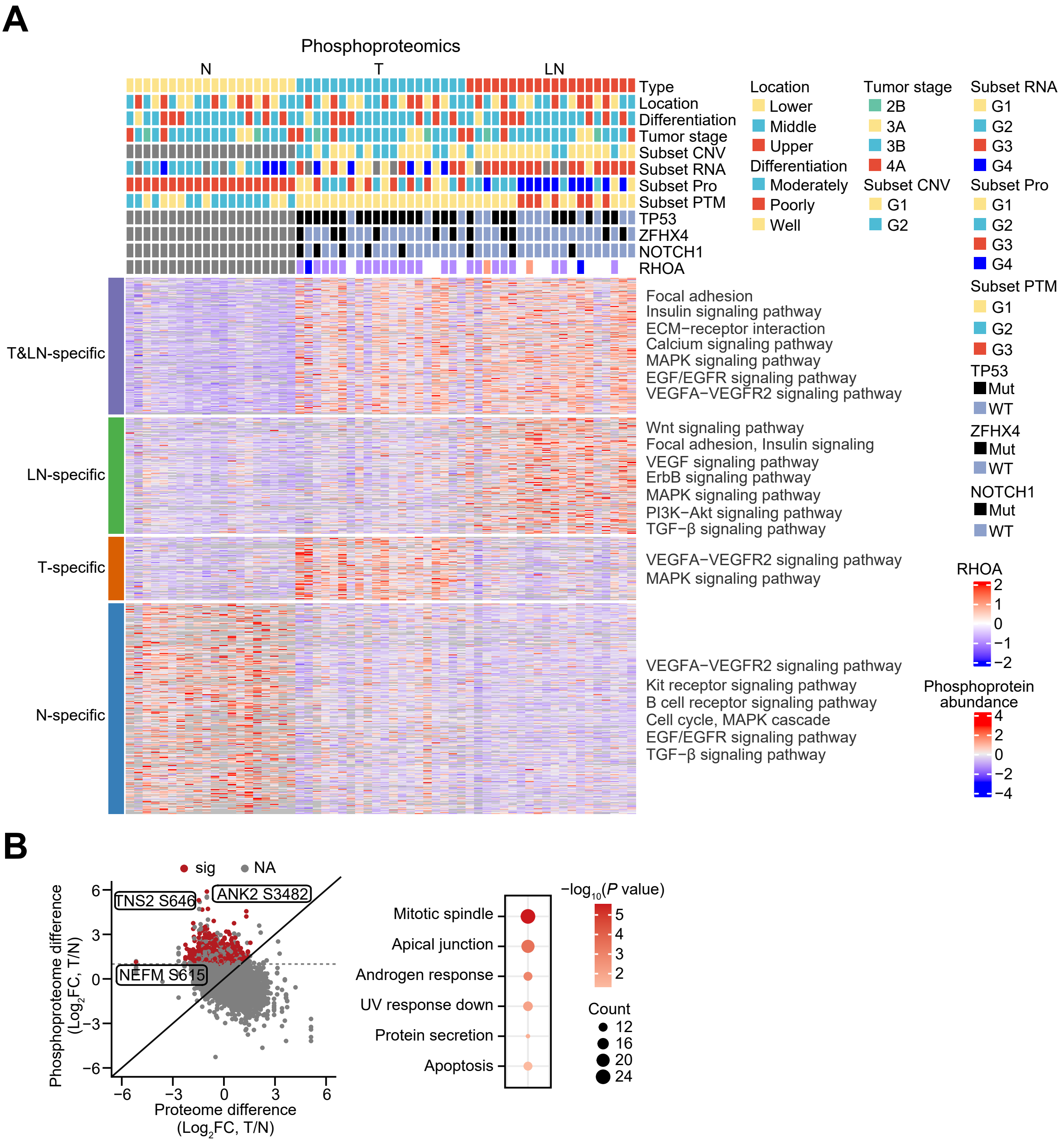

Supplementary Figure 2

Supplementary Fig. 2 Integrative analysis of proteomics and phosphoproteomics data in metastatic ESCC samples

(A) Heatmap showing the differentially expressed phosphoproteins among the four classes. Tiling bars above the heatmap show the distribution of different clinicopathological characteristics among our ESCC cohort. **(B)** FC of proteins and phosphosites, and their correlations in T and N. Pathways enriched with cancer related phosphoproteins.

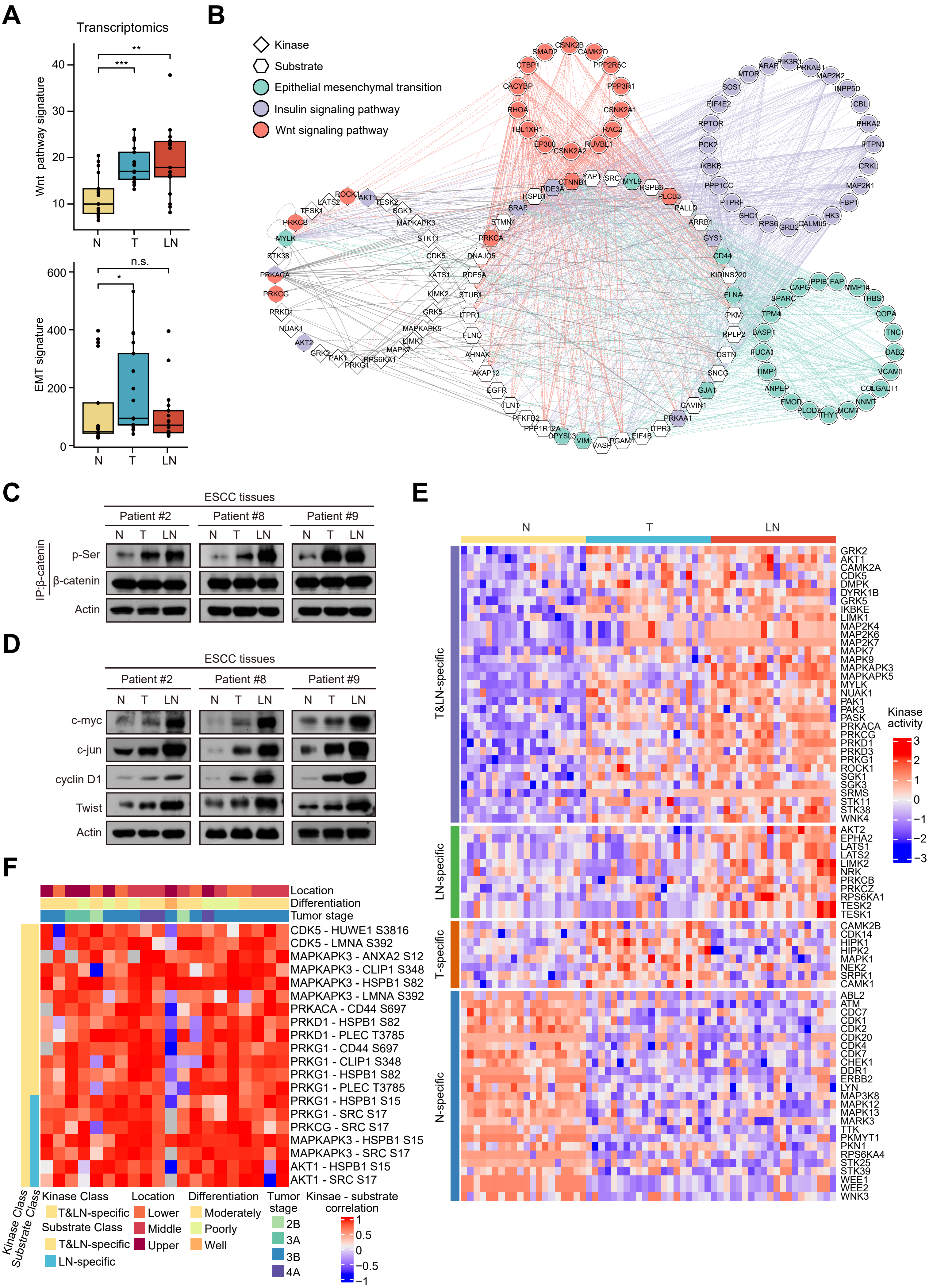

Supplementary Figure 3

Supplementary Fig. 3 Phosphoproteomic profiling in metastatic ESCC cohort

(A) Boxplots showing the distribution of Wnt/β-catenin signaling and EMT signaling at the transcriptome level in N, T and LN samples from ESCC patients. **(B)** Phospho-regulatory network in ESCC. Shown is the network linking cancer-related kinases (diamonds), phosphoproteins (hexagons) and proteins (circles) in oncogenic signaling pathways. The circles in different colors represent the proteins in different oncogenic signaling pathways. **(C)** The phosphorylation level of β-catenin was measured in N, T and LN from ESCC patients. **(D)** Measurement of c-myc, c-jun, cyclin D1 and twist expression in N, T and LN from ESCC patients by Western blot. **(E)** Single-sample gene set enrichment analysis (ssGSEA) was used to estimate the kinase activities. Heatmap showing the differentially expressed kinases among the four classes. **(F)** Kinase-substrate correlations in each sample. Heatmap showed the high-confidence kinase-substrate pairs, the substrates were oncogenes and the kinases were druggable.

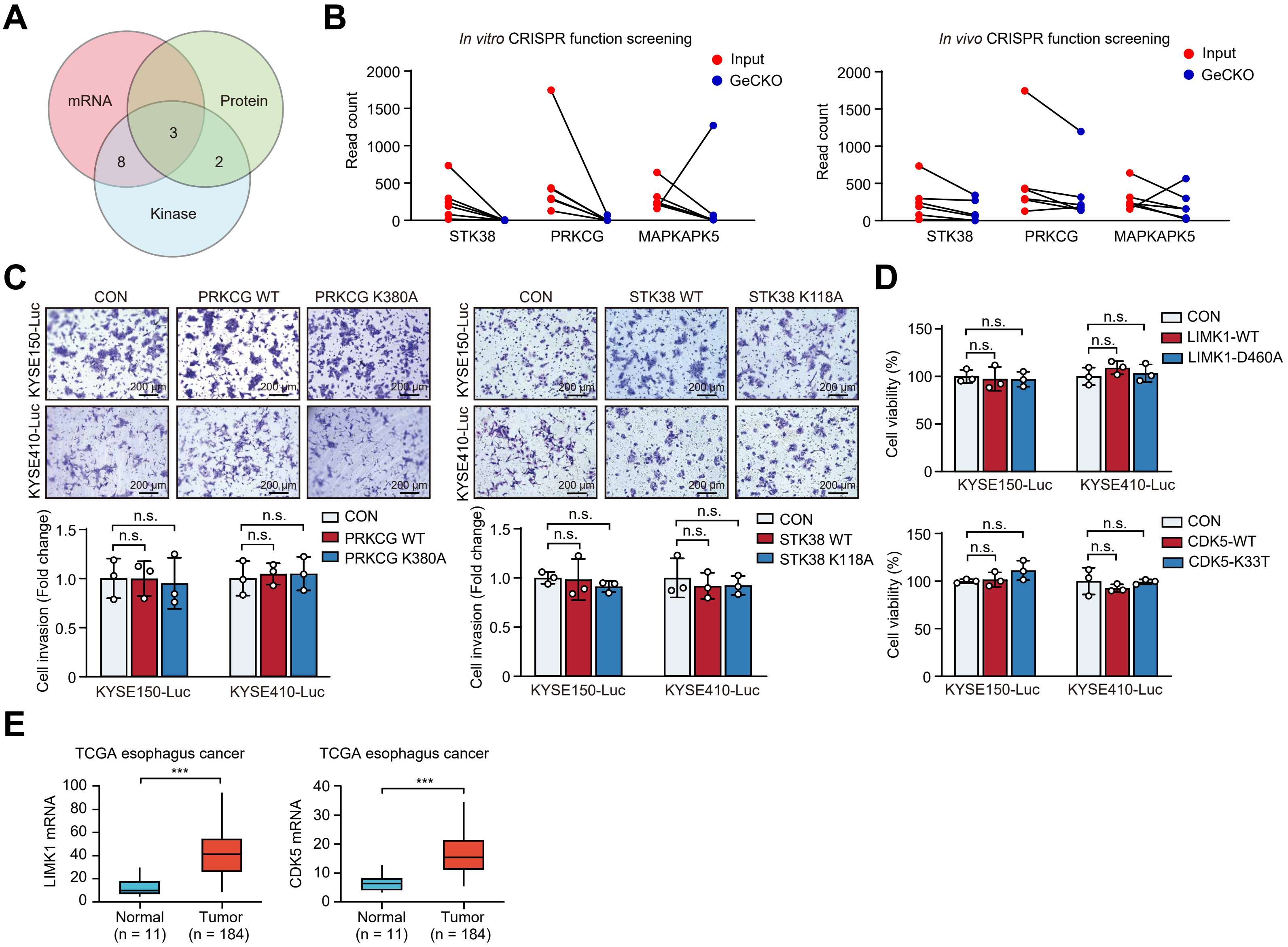

Supplementary Figure 4

Supplementary Fig. 4 LIMK1 and CDK5 are key regulators of ESCC metastasis

(A) Venn diagram shows the overlapping significantly activated kinases and significantly up-regulated genes or proteins in metastatic ESCC. **(B)** Read counts of sgRNAs targeting STK38, PRKCG or MAPKAPK5 in GeCKO-transduced cells and input cells through in vitro (left) and in vivo (right) screening. **(C)** Comparison of the invasion ability of ESCC cells overexpressing wide-type or mutant type of PRKCG and STK38. **(D)** The effect of LIMK1/CDK5 overexpression on the proliferation of ESCC cells was detected using CCK8 assay. **(E)** Boxplots depicting the expression levels of LIMK1 and CDK5 in esophageal cancer cohort from UALCAN database.

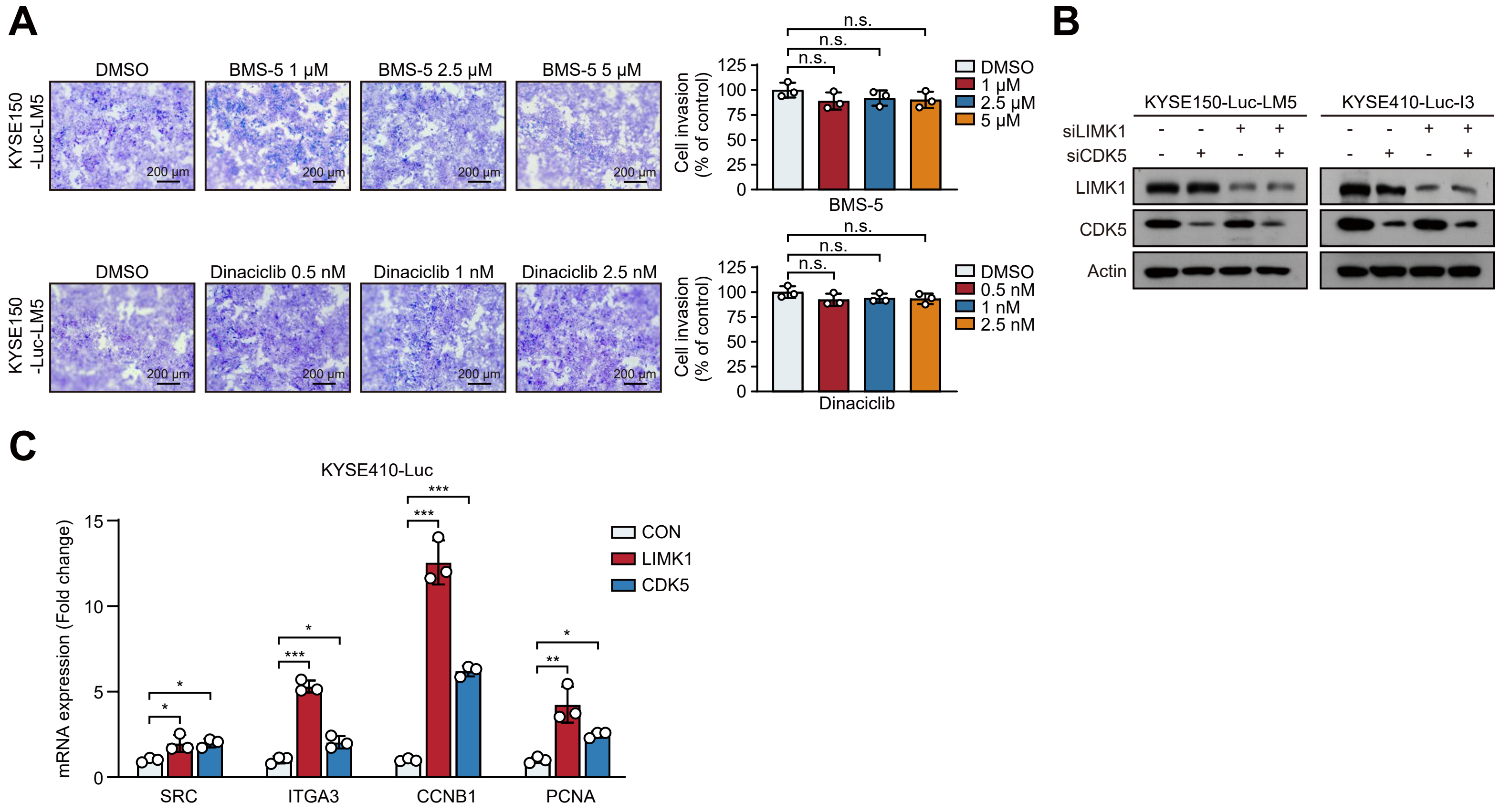

Supplementary Figure 5

Supplementary Fig. 5 LIMK1 and CDK5 synergistically promote the ESCC metastasis

(A) Boyden chamber assay showing the effect of BMS-5 and Dinaciclib on cell invasion at different concentrations. **(B)** Western blot showing the expression of LIMK1 and CDK5 when knockdown via siRNA. **(C)** The mRNA level of SRC, ITGA3, CCNB1 and PCNA are analyzed when LIMK1 or CDK5 is overexpressed in ESCC cell.

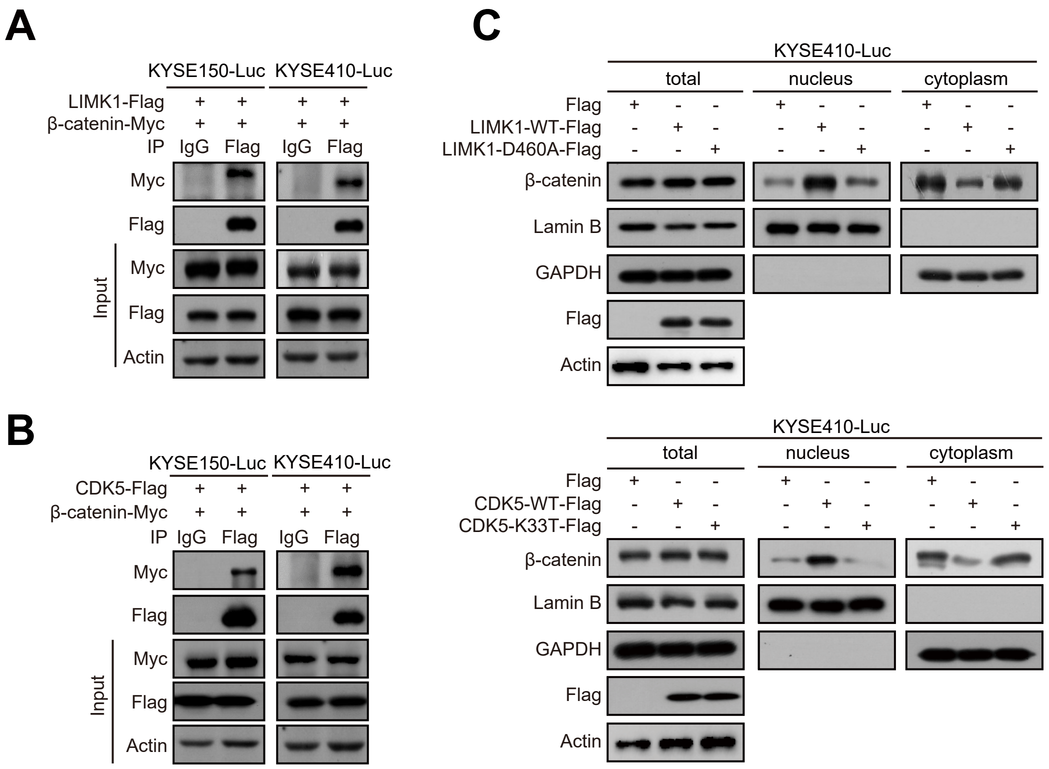

Supplementary Figure 6

Supplementary Fig. 6 LIMK1 and CDK5 interact with β-catenin to promote its nuclear translocation

(A-B) Co-IP assay confirming the endogenous interaction between LIMK1 (A)/CDK5(B) and β-catenin. **(C)** Subcellular fractionation and Western blotting were used to assess the expression of β-catenin in the nucleus and cytoplasm of ESCC cells overexpressing LIMK1-WT/LIMK1-D460A or CDK5-WT/CDK5-K33T.

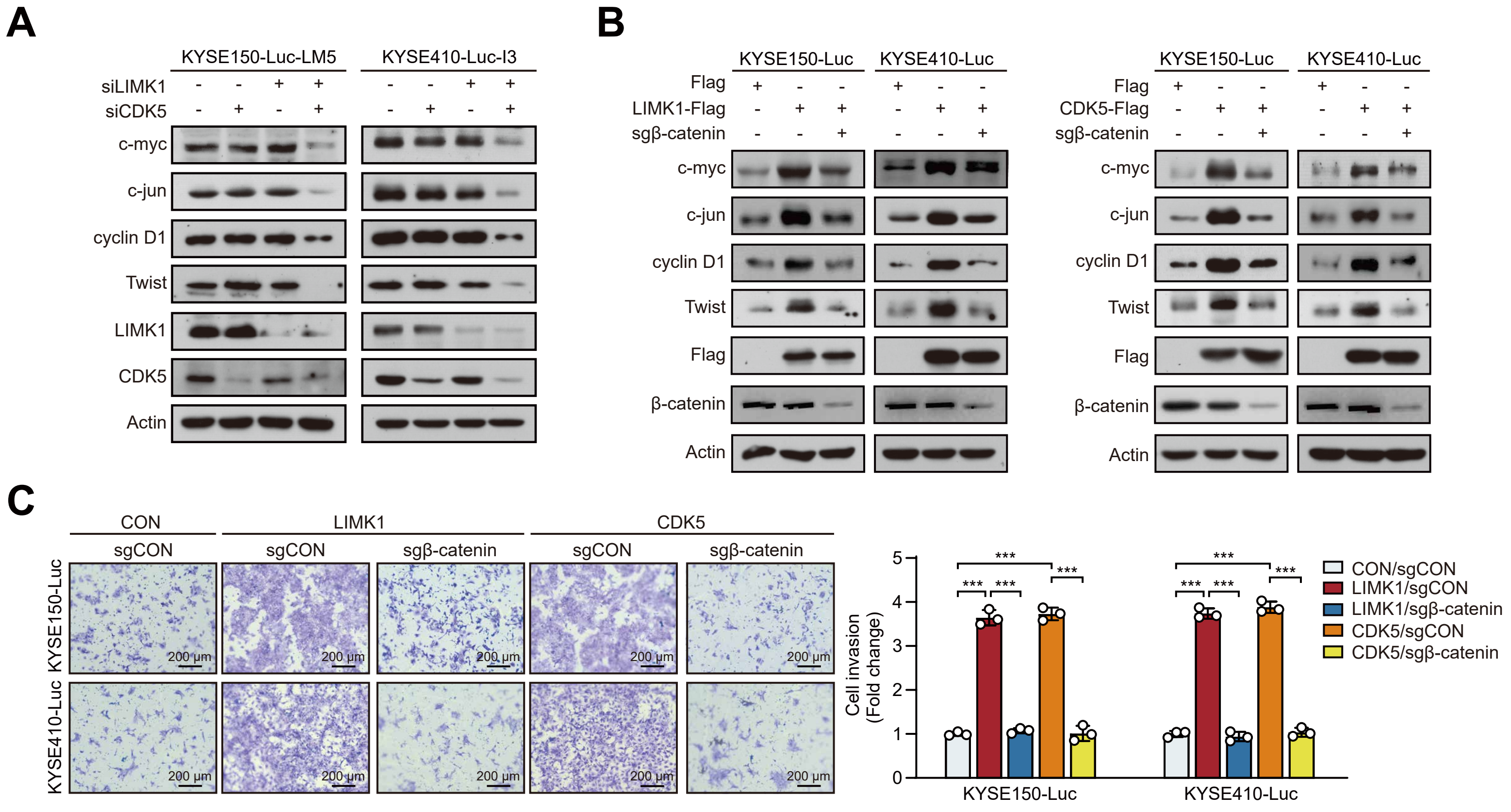

Supplementary Figure 7

Supplementary Fig. 7 β-Catenin mediated the effect of LIMK1/CDK5 on ESCC metastasis.

(A) The effect of LIMK1 and/or CDK5 knockdown on the Wnt signal pathway target genes in ESCC cells is detected using western blot. **(B)** Western blotting was used to detect the expression of downstream proteins in the Wnt signaling pathway in ESCC cells overexpressing LIMK1/CDK5 and with β-catenin knockdown. **(C)** The Boyden chamber invasion assay showed that knockdown of β-catenin attenuated the effect of LIMK1 or CDK5 on cell invasion.

		CDK5		
		Low	High	Total
	Low	50	37	87
LIMK1	High	47	74	121
	Total	97	111	
				P < 0.01

Supplementary Figure 8

Supplementary Fig. 8 LIMK1 and CDK5 expression was significantly correlated.

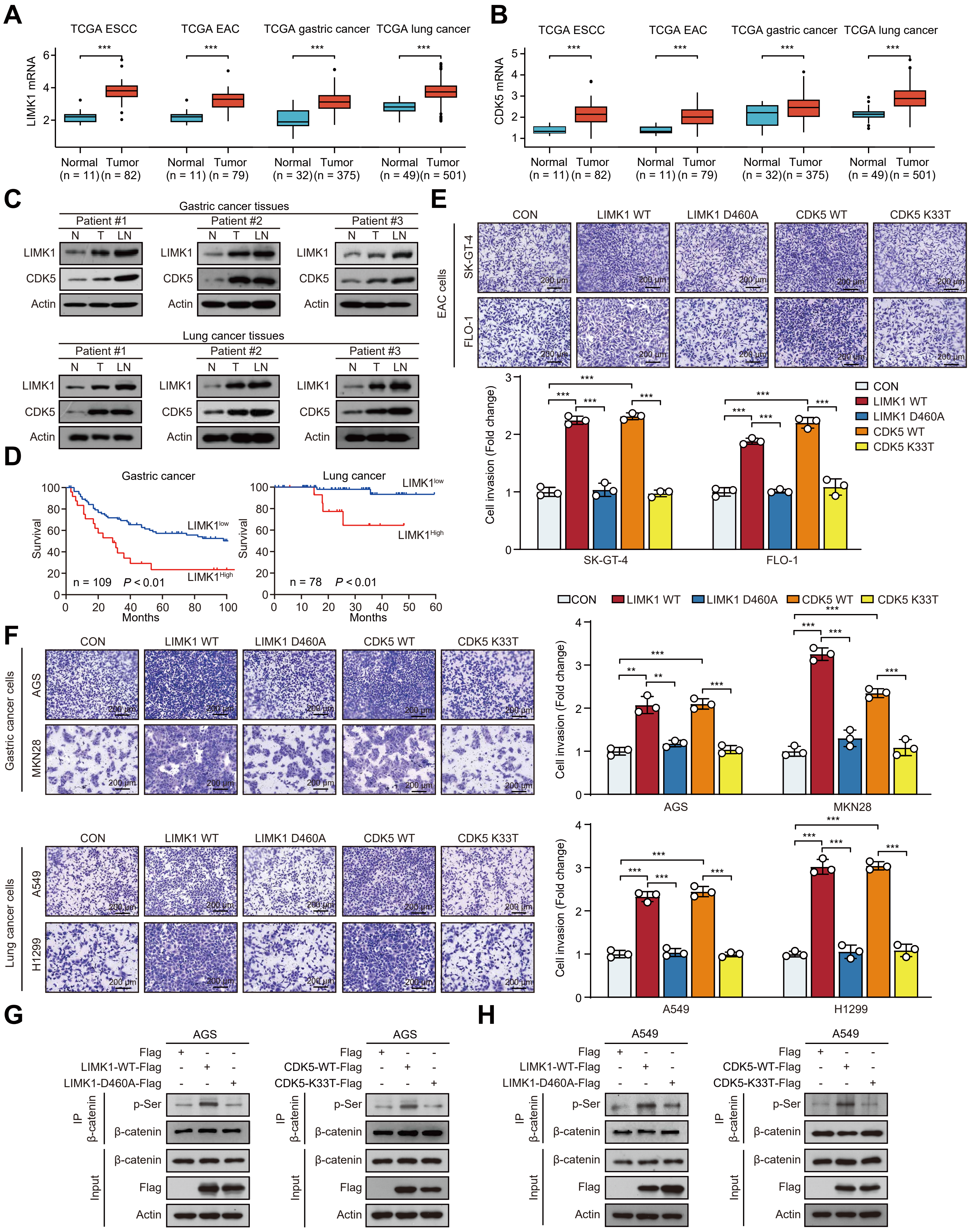

Supplementary Figure 9

Supplementary Fig. 9 LIMK1 and CDK5 promote multiple types of cancer invasion.

(A, B) Boxplots depict the expression level of LIMK1(A) and CDK5(B) in esophagus cancer, gastric cancer and lung cancer from TCGA. **(C)** Expression of LIMK1 and CDK5 in normal tissues (N), matched primary tumor tissues (T) and lymph node metastatic tissue (LN) from gastric cancer and lung cancer patients. **(D)** Kaplan-Meier survival of patients with gastric cancer and lung cancer stratified by LIMK1 expression from TCGA. **(E-F)** LIMK1 and CDK5 positively regulated EAC (E), gastric cancer and lung cancer (F) cells invasion in a manner dependent on their phosphorylation activity. **(G, H)** CO-immunoprecipitation assay showing that LIMK1/CDK5 increases phosphorylation of β-catenin in gastric cancer (G) and lung cancer (H).

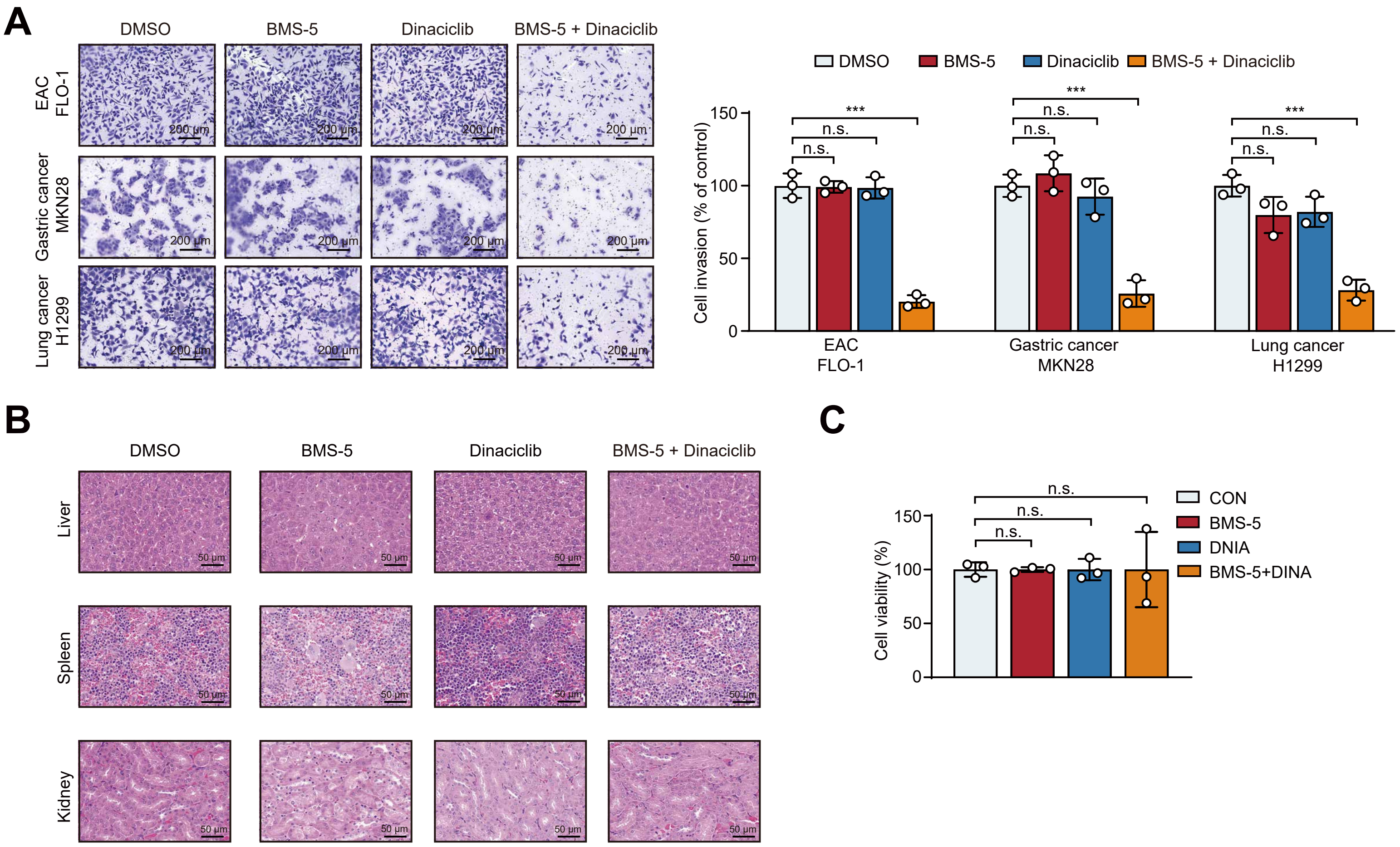

Supplementary Figure 10

Supplementary Fig. 10 Preclinical setting and toxicity assessment of LIMK1 and CDK5 as therapeutic targets for tumor metastasis.

(A) Transwell assays were performed to determine the invasive abilities of EAC, gastric cancer and lung cancer cells when treated with BMS-5, Dinaciclib, or the combination. **(B)**Histological analysis of major organs of mice showing no significant changes among groups. **(C)** The effect of BMS-5 or Dinaciclib on normal esophageal cells proliferation was detected by CCK8.
